# Interventions to promote child development in institutionalized children: a scoping review

**DOI:** 10.1590/1980-220X-REEUSP-2025-0044en

**Published:** 2025-11-17

**Authors:** Vanessa Martins, Vitória da Silva Porto, Katherine Solís-Cordero, Clariana Vitória Ramos de Oliveira, Jéssica Batistela Vicente, Daniela Doulavince Amador, Maria de La Ó Ramallo Veríssimo, Samara Macedo Cordeiro

**Affiliations:** 1 Universidade Estadual de Campinas, Faculdade de Enfermagem, Campinas, SP, Brazil.; 2 Universidad de Costa Rica, Escuela de Enfermería, San José, Costa Rica.; 3 University of Nevada Las Vegas, School of Nursing, Las Vegas, Nevada, United States.; 4 Universidade Federal do Paraná, Coordenadoria de Atenção Integral à Saúde Estudantil, Curitiba, PR, Brazil.; 5 Universidade de São Paulo, Escola de Enfermagem, São Paulo, SP, Brazil.

**Keywords:** Child Development, Child, Institutionalized, Caregivers

## Abstract

**Objective:**

To map the characteristics and results of interventions carried out with caregivers of institutionalized children to promote child development.

**Method:**

A scoping review, following JBI guidelines, was conducted in eight databases, gray literature, and reference lists of selected studies. The review question was: what are the characteristics and outcomes of interventions implemented with caregivers to promote child development in institutionalized children aged 0 to 6 years? Rayyan® software was used to select the studies. The analysis was descriptive.

**Results:**

Seventeen studies were included, reporting training-type interventions, whether or not associated with changes in the institution’s work process and structural organization as well as training through the creation of a virtual learning environment. There were positive effects on child development, but the heterogeneity of interventions and results prevented us from identifying their most relevant aspects. The main content covered was responsive care.

**Conclusion:**

The diversity of professional training models does not allow us to state which aspects are most relevant in the training of caregivers, requiring more robust studies on the subject.

## INTRODUCTION

Early childhood, from 0 to 6 years of age^(1)^, is a period characterized by both vulnerabilities and opportunities^(2)^, during which children experience physical, cognitive, and psychosocial changes, as well as accelerated brain development. This stage represents a strategic time to promote optimal development. Many children may experience this phase while temporarily separated from their families and/or primary caregivers in institutional care until they can be reunited with them or placed with foster families^(3)^. According to the National Council of Justice, around 34 thousand children live in foster care in Brazil^(4)^.

Child development (CD) is an active and unique process for each child, expressed by continuity and changes in the acquisition of motor, cognitive, psychosocial and language skills, and the first years of life are the foundation of this process^(5)^. Care focused on developmental needs enables children to reach their full potential^(6)^.

Promoting children’s development is a priority at national and international levels^(7)^. In Brazil, the Legal Framework for Early Childhood^(1)^ establishes as a priority the creation of programs and public policies that focus on comprehensive development of early childhood, in addition to highlighting the importance of qualifying professionals, who play a fundamental role in providing direct assistance or supporting other caregivers, employing participatory pedagogical techniques that encourage reflection and decision-making regarding behaviors appropriate to the individual needs of each child.

The importance of this topic has been endorsed worldwide by the Sustainable Development Goals (SDGs)^(8)^, the Global Strategy for Women’s, Children’s and Adolescents’ Health (2016–2030)^(9)^, and Nurturing Care, which prioritizes interventions that promote comprehensive early childhood development, including strengthening parenting skills to provide responsive care for their children’s essential needs^(10)^.

Based on knowledge demonstrating that early childhood interactions affect brain circuits and promote emotional and intellectual development^(2)^, CD promotion and child protection and care are no longer seen as the exclusive responsibility of parents and family members, but rather as a broad political and social commitment. This commitment is even more essential in the context of children in residential care, as foster children often experience adverse events involving violence and toxic stress.

Interventions aimed at CD have expanded knowledge in the field of developmental science. Evidence indicates that responsive care, particularly methods that promote caregiver- child interaction, is the most effective intervention for CD^(10,11)^. Responsive care enables us to meet children’s essential needs. These needs include forming healthy and lasting relationships, ensuring physical safety, respecting individual characteristics, providing experiences that align with their developmental stage, and fostering a stable and supportive community^(12)^.

A systematic review^(13)^ found that structural interventions conducted in shelters and training activities with caregivers were effective in promoting CD. Despite evidence indicating the effectiveness of these interventions, the literature lacks detailed descriptions of their characteristics, particularly in the Brazilian context. This gap hinders the replication, adaptation, and scaling of evidence-based practices, which compromises the quality of care provided.

Considering that care in childcare institutions is carried out by lay caregivers, whose minimum required education is high school^(14)^, training on growth and CD becomes necessary.

Educational interventions can enhance the quality of care and relationships between children and their caregivers. This justifies the need to identify and select evidence to develop a program to promote CD in the institutionalized setting of children under six. Therefore, this study aimed to map the characteristics and outcomes of interventions conducted with caregivers of institutionalized children to promote CD.

No studies were found that allow us to understand the characteristics of interventions conducted with caregivers of institutionalized children to promote CD. However, a detailed and comprehensive analysis of these interventions can identify characteristics that favor their implementation, replication, or the development of new studies based on the findings.

## METHOD

### Study Design

This scoping review maps and characterizes the extent of evidence on a field of research, which can highlight knowledge gaps and demonstrate the relevance and need for more in-depth study on certain areas^(15)^. This study was developed and structured according to the JBI recommendations^(15)^, as detailed in the following topics, and the Preferred Reporting Items for Systematic reviews and Meta-Analyses extension for Scoping Reviews (PRISMA-ScR) checklist^(16)^. The review protocol was registered on the Open Science Framework platform (https://doi.org/10.17605/OSF.IO/HN8E2).

This study’s methodological stages were: research question formulation, using the mnemonic Population, Concept and Context (PCC); inclusion and exclusion criteria definition; definition of the types of studies and sources; search strategy development; database identification; study search and selection; data extraction and analysis; and report construction^(15)^.

### Identifying the Research Question

The guiding question was developed based on the PCC mnemonic structure: Population (P) – caregivers; Concept © – interventions to promote CD; Context © – child institutionalization. Accordingly, the guiding question was: What are the characteristics and outcomes of interventions implemented with caregivers to promote CD in institutionalized children aged 0 to 6 years?

Institutionalized children were considered those receiving long-term services, or residing in an institutional environment^(17)^. Interventions to promote CD were actions that promote physical, socio-emotional, cognitive and motor development, implemented for children aged 0 to 6 years. This age range was selected because it corresponds to early childhood, an important phase for a child’s physical, emotional, mental, and social development, making it strategic for promoting CD.

### Selection Criteria

Primary scientific articles and reviews, descriptive or analytical, theses and dissertations that addressed the review question were included. No restrictions were applied regarding time, geographic location, or language.

Studies that did not address the population, concept, and context of interest, or that did not report the implementation of an intervention, as well as editorials, letters to the editor, and opinion pieces were excluded. Furthermore, studies in which the child was under the care of a foster family were excluded. The foster care program, created in 2009, seeks to place children with families selected, trained, and monitored by the municipal government^(18)^. These cases were excluded since the child was not in an institutional setting. Duplicate documents were considered only once.

### Search Strategies

An electronic search was conducted from September to December 2023, updated in April 2025, and assisted by a librarian. The search used Health Sciences Descriptors (DeCS), Medical Subject Headings (MeSH), and the Boolean operators AND and OR in databases and gray literature (Chart 1). A manual search of the reference lists of included articles was also performed.

**Chart 1.** Search strategies according to databases and gray literature – São Paulo, SP, Brazil, 2025.

Database and gray literature	Search strategy
PubMed	(((Child Development[MeSH Terms]) OR (“Child Development”[Title/Abstract] OR “Infant Development”[Title/Abstract])) AND ((Child, Institutionalized[MeSH Terms]) OR (“Child, Institutionalized”[Title/Abstract] OR “Institutionalized Child”[Title/Abstract] OR “Institutionalized Children”[Title/Abstract]))) AND ((Caregivers[MeSH Terms]) OR (Caregivers[Title/Abstract] OR Caregiver[Title/Abstract] OR Carers[Title/Abstract] OR Carer[Title/Abstract] OR “Care Givers”[Title/Abstract] OR “Care Giver”[Title/Abstract] OR “Spouse Caregivers”[Title/Abstract] OR “Spouse Caregiver”[Title/Abstract] OR “Informal Caregivers”[Title/Abstract] OR “Informal Caregiver”[Title/Abstract]))
Virtual Health Library	(“Child Development” OR “Infant Development”) AND (“Child, Institutionalized” OR “Institutionalized Child” OR “Institutionalized Children”) AND (caregivers OR caregiver OR carers OR carer OR “Care Givers” OR “Care Giver” OR “Spouse Caregivers” OR “Spouse Caregiver” OR “Informal Caregivers” OR “Informal Caregiver”)
Scopus	(TITLE-ABS-KEY (“Child Development” OR “Infant Development”)) AND (TITLE-ABS-KEY (“Child, Institutionalized” OR “Institutionalized Child” OR “Institutionalized Children”)) AND (TITLE-ABS-KEY (caregivers OR caregiver OR carers OR carer OR “Care Givers” OR “Care Giver” OR “Spouse Caregivers” OR “Spouse Caregiver” OR “Informal Caregivers” OR “Informal Caregiver”))
Scientific Electronic Library Online	“Child Development” AND “Child, Institutionalized” AND “caregivers”
Cochrane	“Child Development” AND “Child, Institutionalized” AND “caregivers”
Coordination for the Improvement of Higher Education Personnel Journal Portal	“Child Development” AND “Child, Institutionalized” AND “caregivers”
Web of Science	“Child Development” AND “Child, Institutionalized” AND “caregivers”
PsycINFO	“Child Development” AND “Child, Institutionalized” AND “caregivers”
Google Scholar	“Child Development” AND “Child, Institutionalized” AND “caregivers”
Brazilian Digital Library of Theses and Dissertations	“Child Development” AND “Child, Institutionalized” AND “caregivers”

### Study Selection

Search results were imported into Rayyan® for study selection and duplicate removal.The selection process occurred in two phases. In the first phase, two trained independent reviewers read and assessed the titles and abstracts of identified records to preselect potentially eligible studies. In cases of disagreement, a third reviewer decided whether to include the articles. This reviewer was experienced in CD and in conducting systematic literature reviews. In the second phase, one of the reviewers assessed the full text of preselected studies to confirm their eligibility.

### Data Extraction and Synthesis

Data from the included studies were extracted using a standardized form capturing title, author, country, year, design, and objective. The data extracted to characterize the interventions included population, type of intervention, context of intervention, and development domain, in addition to those recommended by the Template for Intervention Description and Replication (*TIDieR*)^(19)^, such as the theoretical framework used, professional responsible for the intervention, intervention content, materials and strategies used, delivery method, dose (number of times, duration, and period), and intervention results. *TIDieR* aims to improve the detailed description of interventions to enable their replicability. The information was entered into a Microsoft Office Word^®^ spreadsheet and allowed for study synthesis and interpretation.

### Ethical Aspects

Since this is review research, the Research Ethics Committee was not required for its development.

## RESULTS

**Figure 1:**
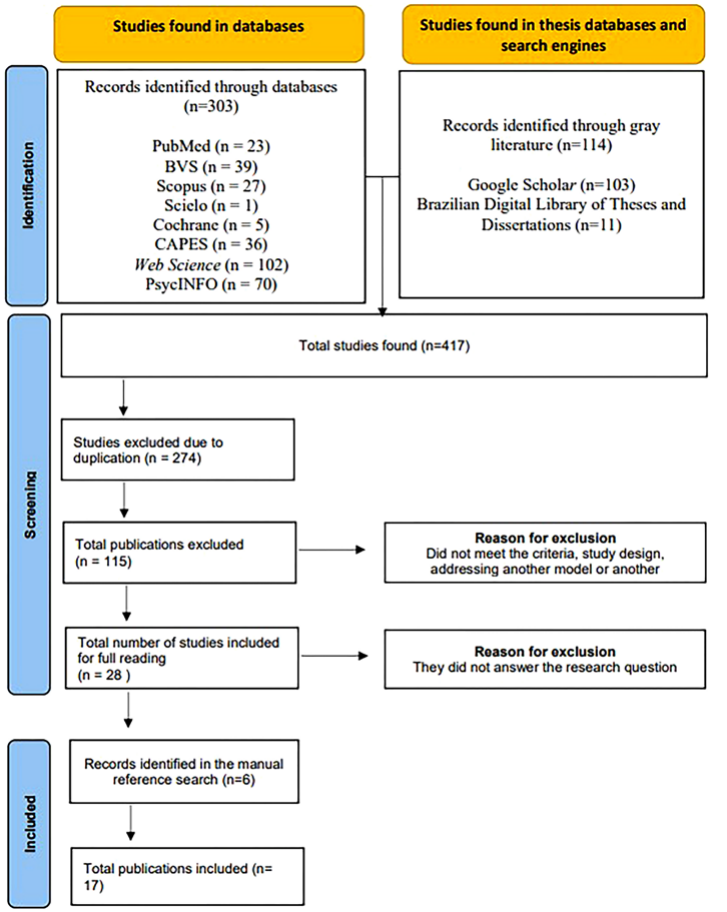
Flowchart of the article selection process for the adapted PRISMA-ScR 2020 flow diagram for new systematic reviews which included searches of databases, records, and other sources. São Paulo, SP, Brazil, 2025.

A total of 417 studies were initially identified. After excluding 274 duplicates using the Rayyan^®^ platform, 143 unique studies remained. Of these, 115 were excluded after assessing the titles and abstracts, leaving 28 that were subjected to full reading. Seventeen articles were excluded during the full reading phase because they did not address the characteristics and outcomes of interventions conducted with caregivers to promote CD for institutionalized children aged 0 to 6 years. Eleven studies were included in the research. A manual search of the reference lists yielded six additional studies, bringing the final sample size to 17 (Figure 1).

Concerning study design, this review identified qualitative studies^(20)^, literature reviews^(13,21,22)^, quasi-experimental intervention studies^(23,24)^, cohort studies^(25,26)^, methodological studies^(27)^, book chapters^(28)^, non-randomized intervention studies^(29,30)^, prospective longitudinal studies^(31)^, pilot interventions^(32,33)^, case studies^(34)^, and feasibility studies^(35)^. The studies were published between 2002 and 2021 and carried out in Brazil^(20,27)^, the United States^(13,21,22,28,32)^, Russia^(23,25,26,29)^, Chile^(24)^, India^(31)^, Turkey^(30)^, Nepal^(34)^, Tanzania^(35)^, and Central American countries^(33)^.

Chart 2 presents the objectives and results of the interventions.

**Chart 2.** Identification of studies by author, country, year of publication, study objective, and results – São Paulo, SP, Brazil, 2025.

Title	Author Country Year	Objective	Results
Cuidadoras de crianças institucionalizadas: intervenção e cuidados	Jacqueline Müllich Fensterseifer Brazil 2021	Present the knowledge produced on how care is provided to babies in foster care.	The interventions helped promote better-quality emotional bonds between caregivers and children. There were improvements in the mental health and well-being of both caregivers and children.
Early Caregiver–Child Interaction and Children’s Development: Lessons from the St. Petersburg-USA Orphanage Intervention Research Project	Robert B McCall *et al*. USA 2018	Conduct a review of studies addressing the development of children residing in institutions in the Russian Federation or placed with families in the United States and Russia.	Improved interactions between caregivers and children in institutions positively impact children’s physical and behavioral development. Caregivers improved their caregiving response to children’s crying.
Caregiver-child Interaction, Caregiver Transitions, And Group Size As Mediators Between Intervention Condition And Attachment And Physical Growth Outcomes In Institutionalized Children.	Hilary A Warner *et al*. Russia 2017	Describe a secondary analysis of data from a Russian intervention study to determine whether the quality of caregiver-child interaction, transitions between caregivers, and group size influence the effect of the intervention on children’s attachment and physical growth.	There was an improvement in the quality of interactions between caregivers and children. Children living in institutions undergoing the intervention had significantly higher height, weight, and head circumference than children who did not undergo the intervention.
Effects of an intervention to promote socioemotional development in terms of attachment security: a study in early institutionalization in Chile	Felipe Lecannelier *et al*. Chile 2014	Assess the psychoaffective state of infants in institutional settings in the areas of attachment, psychomotor development, temperament, and social development, in addition to analyzing the effectiveness of a program to promote socioemotional development implemented in residential centers, considering attachment security as an organizing variable.	There was an improvement in the emotional relationship between the child and the caregiver, in the attention span and in the security of attachment, with an increase in the percentage of babies with secure attachment.
Fostering Child Development by Improving Care Quality: A Systematic Review of the Effectiveness of Structural Interventions and Caregiver Trainings in Institutional Care	Katharin Hermenau *et al*. USA 2016	Systematically review current evidence on the effectiveness of structural interventions and caregiver training in institutional care to promote children’s development by improving the quality of care and preventing maltreatment.	The quality of childcare, especially in the early years of life, has a crucial impact on healthy mental development and functioning later in life. There have been beneficial effects on the emotional, social, and cognitive development of children in institutional settings.
Caregiver sensitivity and consistency and children’s prior family experience as contexts for early development within institutions	Brandi N Hawk *et al*. Russia 2018	Examine the association between caregiver sensitivity and consistency with the socioemotional and cognitive development of institutionalized young children.	Improving caregiver sensitivity and consistency can have a positive impact on the cognitive and socio-emotional development of institutionalized children, especially in the first year.
Maintaining a Social-Emotional Intervention and its Benefits for Institutionalized Children	Robert B McCall *et al*. Russia 2013	Report the maintenance of a socio-emotional intervention carried out in Russian orphanages using the regular caregiver team.	The training and structural change intervention showed developmental improvements during and after the intervention; however, staff turnover can hinder its continuity and affect caregiver consistency, impacting children’s developmental outcomes.
*Estimulação de linguagem para cuidadores de crianças institucionalizadas: elaboração de um blog*	Maria Gabriela Cavalheiro; Simone Aparecida Lopes-Herrera Brazil 2019	Develop a virtual learning environment in the area of language stimulation aimed at training caregivers of institutionalized children in shelters in a Distance Education context.	The results highlighted the potential of Distance Education actions in training professionals linked to the population at risk for language disorders.
Earlier is better: a meta-analysis of 70 years of intervention improving cognitive development in institutionalized children	Marian J. Bakermans-Kranenburg, Marinus H. Van IJzendoorn, Mulher Juffer USA 2008	Review intervention studies aimed at improving the development of institutionalized children.	Interventions conducted in institutions demonstrate significant improvements in children’s cognitive, language, and socio-emotional development. Furthermore, some interventions have also benefited their physical health, growth, and motor development.
Young Children in Institutional Care: Characteristics of Institutions, Children’s Development, and Interventions in Institutions	Megan M Julian *et al*. USA 2019	Describe the social environments of institutions around the world and interventions carried out in these locations.	Improving the quality of care in institutions can have significant impacts on the development of children living there, especially when caregivers regularly interact with the same children, thus increasing their sensitivity and responsiveness during daily activities.
The role of transitions to new age groups in the development of institutionalized children	Robert B McCall *et al*. Russia 2012	Describe the effects of transitioning to new age groups and caregivers on the overall development of institutionalized children.	Rotation of rooms, caregivers, and groups can be detrimental to institutionalized children, but it is mitigated by sensitive caregiver-child interaction. Training to improve this interaction and avoid transitions has led to more positive and less stressful development.
‘Not by bread alone’: impact of a structured 90-minute play session on development of children in an orphanage	V. Taneja *et al*. India 2002	Develop a structured gaming intervention program.	Children showed greater responsiveness, improved head and body control, and were more active. Caregivers were reluctant at the beginning of the intervention to the increased workload, but by the end, they were enthusiastic.
A socioemotional intervention in a Latin American orphanage	Robert B McCall *et al*. USA 2012	Assess how a socioemotional intervention that emphasized training and technical assistance could affect the development of institutionalized children.	After the intervention, there was a significant improvement in interactions between caregivers and children, with a warmer, more attentive, sensitive, and engaging approach, especially for younger children. Improvements were most pronounced during activities such as feeding, bathing/changing clothes, and free play.
Environmental Enrichment and Caregiver Training to Support the Development of Birth to 6-Year-Olds in Turkish Orphanages	Sibel Kazak Berument Turkey 2013	Develop an intervention program that integrates environmental enrichment and caregiver training to improve the language and cognitive development of infants and young children living in institutional settings in Turkey.	The intervention program was effective in increasing the quality of care in orphanages, also having an effect on the linguistic and cognitive development of infants and preschoolers in this study.
From maid to mother: transforming facilities, staff training, and caregiver dignity in an institutional facility for young children in Nepal	Amy Conley Wright *et al*. Nepal 2014	Report the case of improving the health, safety and development of children aged 0 to 6 years in an orphanage in Nepal.	There was a decrease in infectious diseases and an increase in social interactions between children and caregivers. Furthermore, there was progress in children’s cognitive and emotional development after the interventions were implemented, along with an improvement in self-esteem and a sense of “worthiness” among caregivers.
Improving care quality and preventing maltreatment in institutional care – a feasibility study with caregivers	Katharin Hermenau *et al*. Tanzania 2015	Test the feasibility of an intervention to promote CD and assess changes in child maltreatment rates.	Training is feasible even in challenging circumstances. The content covered is applicable to the caregiver’s work process. The children also reported improvements in care delivery.
The Effects of a Social–Emotional Intervention on Caregivers and Children With Disabilities in Two Central American Institutions	Christina J Groark *et al*. Central America 2013	Report a pilot project intervention to improve two Central American institutions serving children with severe and multiple disabilities using a train-the-trainer approach to encourage typical staff caregivers to provide sensitive, responsive, and respectful interactions with children.	Intervention that prioritizes sensitive and responsive care, along with some basic specialized positioning and handling techniques, can be successfully applied to improve the physical and behavioral/mental development of institutionalized children with severe and multiple disabilities.

Legend: USA – United States of America.

All included studies involved caregivers of institutionalized children, with the majority conducted in the institutional care setting. In one study, caregivers received training in a primary school in the region^(35)^. One study reported a virtual intervention^(27)^, 13 reported-in person interventions^(13,20,21,22,23,24,25,26,29,30,31,33,35)^, and three did not describe this information^(28,29,34)^. Among the in- person interventions, five were carried out in groups^(20,24,29,33,35)^, and eight^(13,21,22,23,25,26,28,30)^ did not specify whether they were conducted groups or individually. Two studies mention that, in addition to carrying out group training, individual supervision of caregivers was carried out after the training^(25,33)^.

In relation to the use of theoretical frameworks for constructing the intervention: one study^(20)^ used documents from the health, social psychology, psychoanalysis and social assistance fields, the Statute of Children and Adolescents, and the Technical Guidelines for Child and Adolescent Care Services of 2009; three^(24,25,29)^ used John Bowlby’s Attachment Theory; one^(31)^ used the Social Learning Theory; one^(32)^ used an already known program for child care in association with precepts of Pikler Institute of Budapest; one^(35)^ followed American Academy of Pediatrics (1999) guidelines and pre-established training concepts; and ten studies^(13,22,23,25,36,27,28,30,33,34)^ did not mention a theoretical framework in the intervention elaboration.

The interventions reported fell into three categories: caregiver training focused on promoting CD during care^(13,20,24,33)^; training associated with changes in the institution’s work process and organizational structure^(21,23,25,26,28,29,30,32,34)^; and training delivered via a virtual learning environment^(27)^. One of the studies used a modeling or imitation technique, in which caregivers were encouraged to repeat the intervention agents’ actions^(31)^, and one of the studies, in addition to training and structural changes, offered technical assistance to monitor caregivers^(32)^.

The interventions focused on the socio-emotional development^(13,20,21,22,23,24,25,26,28,29,32,34,35)^, motor^(21,23,26,31,32)^, cognitive^(13,21,22,23,26,30,31,32,33,34)^, language^(20,22,27,30,31,32)^ and physical^(33,34)^ domains.

The materials used for the intervention varied and included: cards and everyday objects from a children’s routine^(20)^, toys and support materials such as changing tables and beds^(33)^, a handbook of attachment sensitivity^(24)^, toys, books and music relevant to each age group^(13,26,30,31,32)^, electronic web record with theoretical and practical content^(27)^, books, and DVDs^(29)^. Seven studies did not describe the materials used in the intervention^(21,22,23,25,28,34,35)^.

The professionals responsible for the implementation of the intervention were psychologists^(20,31)^, psychologists with the support of nurses^(35)^, a multidisciplinary team with the presence of nurses^(21,25)^ and speech therapists^(27)^. In one study, the intervention was delivered by professionals from two companies focused on CD—one of them non-profit—with additional support from a social worker and a psychologist^(29)^. Seven studies did not specify the professional responsible for conducting the intervention^(13,22,23,24,26,28,29)^. One study only reports that the professional received prior graduate training to implement the intervention^(33)^.

The studies varied in the distribution of their interventions regarding the dose. Four studies did not describe the period of implementation, duration, or frequency^(23,26,28,29)^. Research carried out the intervention in 90 hours of observation and workshops held in four meetings^(20)^. Other interventions varied from 12 to 14 meetings^(21)^; training was carried out in four hours^(24)^; there were trainings with nine modules and 11 sessions of three hours for each module^(25)^; there was a year of weekly training with a duration of three hours per session^(34)^; there was training for two weeks, eight hours a day and six days a week^(35)^; and there were nine monthly sessions of eight hours, complemented by monthly mini sessions of one to three hours^(33)^. An intervention was divided into two phases, the first occurring over one month and the second over two months, and each session within these phases occurred over 90 minutes^(31)^. Another intervention occurred weekly over a period of one year^(32)^. An intervention was divided into training that took place over 17 weeks with biweekly meetings lasting 90 minutes each and an activity supervision phase, in which eight meetings were planned, but only two were carried out^(30)^.

The main content of the interventions focused on was the provision of love, sensitive and responsive care^(21,23,25,26,28,29,30,32,33)^, establishment of a secure attachment between babies and their caregivers^(24,29,30)^, prevention of abuse^(35)^, child language acquisition, and the importance of caregivers in this process^(27)^. Moreover, preschool children were encouraged with learning topics such as colors, numbers, and seasons^(30)^. Another focus of the intervention was to improve daily work and care skills^(34)^.

Changes to the work process included reducing the number of caregivers per group of children, reducing the number of children per caregiver, increasing consistency of care from one primary caregiver, and creating “family time”, which is quality time without distractions or interference between caregivers and children^(21,23,26,28,29)^. One of the studies provided improvements to the menu, hygiene of materials and sanitation, and also instituted regular visits by physicians and nurses for healthcare^(34)^.

## DISCUSSION

This scoping review revealed that interventions designed to train caregivers of institutionalized children can significantly improve their socio-emotional, motor, cognitive, linguistic, and physical development.

The use of different types of interventions can lead to varying degrees of success in implementation. Studies show that some training methods, such as unsupervised in-person workshops, do not increase caregiver skill acquisition^(36,37)^, while more intensive training methods can lead to significant gains in provider competencies.

A study assessing therapist training in psychosocial skills found evidence that workshops with additional guidance and “multi-component” training that included observation, feedback, and coaching increased skill acquisition^(36)^. However, it remains unclear whether more rigorous training methods that can improve outcomes for caregivers also improve outcomes for the population they care for^(38)^.

A study conducted in Australia with occupational therapists identified a preference for individual post-training support to implement the taught program in practice, confirming data from the literature that indicates that providing new information alone has a limited impact on behavior change^(39)^.

Another study conducted with school assistants demonstrated that combining a contextual approach with individual coaching after a workshop is more effective than conducting a workshop alone at improving these professionals’ performance^(40)^. Coaching is defined as an interactive learning process in which an expert observes another individual’s performance and provides objective, formative feedback to promote self-reflection on performance and achieve previously established goals^(41)^.

Studies show that monitoring caregiver activities and behaviors, in addition to encouraging the adoption of qualified practices, is crucial for the sustainability of the intervention and changes in CD promotion^(32)^.

Sustainability refers to the continuity of the positive effects of interventions and the maintenance of their long-term effects^(42)^. According to the literature, other factors that influence sustainability include adaptability, or the ability to change depending on the context over time, and organizational learning, which involves continuing training and low staff turnover. Furthermore, organizational support and partnerships are essential to creating a favorable internal environment. Integrating the intervention with the institutional mission and having “advocates” who can sustain it during vulnerable periods are also crucial. Additionally, funding must be continuous and adequate, coming from diverse sources. The maintenance of benefits depends on participants’ perception that the intervention is achieving the desired initial results^(42)^. A systematic literature review shows that interventions that include training components (both professional and non-professional) tend to be more sustainable than those that do not incorporate such activities. Trained individuals have the ability to continue providing benefits by training community members, establishing a support network for the program^(43)^.

Among the analyzed interventions, caregiver training combined with structural changes stands out. The results indicate improvements in the quality of caregiver-child interactions, reduced rates of developmental delays, and increased awareness of the importance of responsive care in these institutions. These findings highlight the effectiveness of interventions that integrate training with structural modifications, offering a robust basis for future initiatives aimed at fostering CD.

Furthermore, these studies emphasize the continued need for investment in caregiver training programs to improve the quality of care provided to children^(44)^. A study conducted in Tanzania with children under 18 months of age found that interventions that encourage training and active participation of caregivers and children, targeting CD, had a significant impact on cognitive and linguistic development^(44)^. Evidence that interventions that combine training with structural changes result in significant improvements in caregiver-child interactions and CD highlights the importance of integrated approaches and multicomponent interventions in promoting child well-being^(44,45)^. However, it is important to acknowledge that the success of these interventions hinges on more than just effective implementation; it also requires the continuous support and engagement of the communities and institutions involved. Therefore, future research and policymaking should focus on ensuring the sustainability of these programs and adapting them to different cultural and social contexts.

When developing interventions with caregivers, among the studies that presented a theoretical framework, the most frequently used theory was John Bowlby’s Attachment Theory. Bowlby considered attachment an innate human mechanism, with individuals biologically programmed to recognize the presence of an available figure who offers a relationship of exchange and provides a sense of security^(46)^. Typically, the child’s attachment figure is the biological mother. However, this paradigm is broken in the case of institutionalized children due to a rupture of family ties, either momentary or continuous. Thus, caregivers begin to exercise this role^(46)^.

Children need specific resources to achieve their intellectual, social, emotional, and physical potential, which are provided through the fulfillment of their essential needs^(12)^. The first need is for continuous, supportive relationships. The presence of a primary caregiver and the opportunity for constant interaction ensure the development of emotional security. Promoting sensitive care and secure attachment contributes to CD.

Training sessions to promote CD with caregivers cover topics such as the relevance of the caregiver-child relationship, establishing a secure bond, providing responsive care, and preventing abuse. Sensitive and responsive interactions between caregivers and children are fundamental to the experiences of children in institutions and can contribute to their physical growth and development^(47)^. The content discussed with caregivers is according to the Nurturing Care strategy, launched by the World Health Organization in 2018. This strategy highlights the importance of global actions aimed at responsive care for children and recognizes CD as an important element in achieving the SDGs – 2030^(10)^. The Nurturing Care model guides the actions needed to promote comprehensive development at all stages of childhood, especially in early childhood. According to this model, children require five interrelated components of care: good health, adequate nutrition, safety and security, responsive care, and learning opportunities. Responsive care is defined as caregivers’ ability to perceive, understand, and appropriately and promptly respond to children’s cues to meet their needs^(48)^.

Among the studies analyzed that carried out the intervention to promote CD in person, eight of them did not report whether the action was carried out individually or in a group^(13,21,22,23,25,26,30,31)^, while the five were conducted in a group^(20,24,32,33,35)^. Furthermore, two studies associated individual supervision with group training^(25,33)^, suggesting a preference among researchers for group approaches. This corroborates current research showing the prevalence of group approaches to discussing the topic with parents and caregivers. These approaches facilitate knowledge construction among participants through interaction, sharing experiences, and expressing difficulties and concerns^(49,50)^.

Notably, there is a lack of information about the intervention materials used in the interventions, since this data was only available in two studies: one that shared the training course guide and the cards previously used to raise awareness among caregivers^(20)^; and another that inserted images of the final configuration of the blog with information for caregivers^(27)^. This lack of information makes it difficult to analyze the materials used and prevents the interventions from being replicated in other contexts to further assess their effectiveness.

The intervention dosage varied across studies. Five studies lacked descriptions of the intervention’s period, duration, and frequency. The shortest reported intervention period was a workshop held over four meetings^(20)^, while the longest intervention period occurred from 2004 to 2008^(34)^. The duration of the educational workshops varied. The shortest session was 90 minutes^(31)^, and the longest session was eight hours^(35)^. Although there is this lack of uniformity in the description, it is important to emphasize that all studies reported positive results of interventions in the context of CD.

The variation in intervention dosage observed across studies underscores the importance of a standardized, detailed approach to describing intervention timing, duration, and frequency. This uniformity is important because it allows for comparisons across studies and a better understanding of results^(45)^. Furthermore, a standardized approach aids in the replicability of interventions, allowing other researchers to implement and assess the same strategies in different contexts^(51)^. This advances scientific knowledge about effective CD interventions, enabling the identification of best practices for promoting children’s well-being.

According to the “Paths and Learning for Initiatives Focused on Early Childhood” manual, published by *Núcleo Ciência Pela Infância* (NCPI), active and empathetic listening to the demands, desires, needs, and perceptions of the intended recipients of an intervention is necessary for its development. Field visits and immersions are favorable approaches for being closer to the reality and particularities of the context and learning from the target audience^(52)^. The NCPI is an inter-institutional coalition that includes national and international research centers. These centers include the Center on the Developing Child at Harvard University, the Institute of Education and Research, the *Fundação Maria Cecília Souto Vidigal*, and researchers from the *Universidade de São Paulo*. The NCPI works closely with policymakers and public administrators. Although it does not have normative status, the aforementioned manual has been widely referenced in training courses, government programs, and third- sector initiatives, due to its empirical basis and applicability in different contexts. The manual is aimed particularly at health, education, social assistance, and urban planning professionals who work to promote comprehensive CD.

Additionally, the IDEAS Impact Framework methodology, proposed by the Frontiers of Innovation of the Center on the Developing Child at Harvard University (2017)^(53)^, structures research approaches that ultimately aim to achieve large-scale interventions. In other words, these interventions must reach more people and be self-sustaining to achieve sustainability^(53)^. According to the methodology, it is recommended that the intervention be initially implemented in a small group of the target population. With a smaller sample, it becomes easier and faster to conduct testing cycles to collect data and verify the intervention’s practicality. These cycles are essential for obtaining important lessons^(53)^. This method is relevant in the field of implementation science because it can guide the development and assessment of evidence-based early childhood interventions. The method’s iterative approach ensures effectiveness, scalability, and sustainability. Additionally, it incorporates systematic data collection and analysis, promoting alignment with the specific needs of the target audience and continuous improvement of change mechanisms^(53)^.

Another important aspect of creating interventions aimed at promoting CD is “co-creation”, i.e., creating the initiative together with those who will use it. Strategies for listening, engaging, and interacting with users help gain a deeper understanding of this audience’s perspective^(53)^.

As recommended by *TIDieR* ^(19)^, the data collected to characterize the interventions reveal a gap in the detailed description of the interventions performed. This lack of information makes it difficult to replicate the study in other contexts. A scoping review that mapped interventions^(54)^ also identified this situation, which stems from the fact that interventions are designed to meet specific objectives rather than being planned as replicable interventions.

A central finding of this review was the significant heterogeneity of the analyzed interventions, in terms of both content and format and duration. This methodological diversity, combined with the lack of information regarding the materials used, dosage of interventions, and implementation strategies, limits the ability to assess the effectiveness of these approaches and hinders their replication and scalability. The lack of standardization weakens the evidence base and represents an obstacle to formulating public policies based on robust data. Therefore, future studies must adopt more systematic and detailed descriptions of interventions, as recommended by instruments such as *TIDieR*, to make them effectively replicable, adaptable, and sustainable in various institutional contexts.

This review’s results demonstrate that caregiver training, structural changes in care environments, and changes in caregivers’ work processes positively impact children’s emotional, social, and cognitive development. The results also highlight the importance of improving the quality of care in child care institutions.

The study’s main contributions stem from its presentation of positive results across all investigations. Furthermore, the study emphasizes the importance of intervention study reports providing more comprehensive descriptions of the intervention itself to ensure replicability and scalability in other settings.

The study has some limitations that should be considered. As a scoping review, it draws data from several studies with different methods and designs, some of which may lack adequate scientific rigor. Additionally, the lack of a detailed description of the essential elements of the interventions makes it difficult to understand and compare the results. The low number of publications is another limitation. Despite efforts to cover all the literature, some studies may have been unintentionally missed, which could influence the final conclusions and summary of the evidence presented.

## CONCLUSION

Interventions conducted with caregivers of institutionalized children to promote CD were conducted through training and instruction associated with structural changes in services, predominantly in-person and in groups. Topics covered in the interventions included providing warmer, more sensitive, and responsive care; establishing a secure attachment between infants and their caregivers; preventing maltreatment; child language acquisition; and the importance of caregivers in this process. The interventions also addressed ways to encourage preschoolers with learning topics. These interventions yielded favorable results and positive effects on CD.

However, significant heterogeneity was observed among the interventions analyzed, with variations in content, format, and duration, in addition to a lack of information on the materials and strategies used. This lack of standardization makes it difficult to identify the most effective interventions, limiting replication and weakening the evidence base for public policy.

Mapping interventions conducted with caregivers of institutionalized children partially addresses the need to identify relevant characteristics for developing future intervention programs to promote CD. Therefore, we suggest conducting further studies to provide robust evidence on the characteristics and outcomes of interventions conducted with caregivers of institutionalized children to promote CD and guide practice. It is also important to highlight the need for the development and gradual implementation of high-quality, sustainable and replicable interventions with caregivers of institutionalized children, in order to strengthen institutional structures and help children living in these spaces have greater opportunities to reach their maximum development potential.

## Data Availability

All data supporting the results of this study were published in the article itself. They were extracted from publicly available articles and sources, as detailed in the method section, and are available in the table of studies included in the review.

## References

[1] O’Connor G, Fragkiadaki G, Fleer M, Rai P (2021). Early childhood science education from 0 to 6: a literature review. Educ Sci.

[2] Clark H, Coll-Seck AM, Banerjee A, Peterson S, Dalglish SL, Ameratunga S (2020). A future for the world’s children? A WHO-UNICEF-Lancet Commission. Lancet.

[3] Bernardi DCF (2020). Levantamento Nacional sobre os serviços de acolhimento para crianças e adolescentes em tempos de Covid-19: apresentação dos resultados.

[4] Brasil, Conselho Nacional de Justiça (2025). Sistema Nacional de Adoção e Acolhimento.

[5] Tancredi CCR, Silva JP, Silva KC, Schnorr MM, Santos MN, Santos R A (2022). O desenvolvimento infantil. Rev Ibero-Am Human Ciênc Educ.

[6] Babik I, Cunha AB, Lobo MA (2022). A model for using developmental science to create effective early intervention programs and technologies to improve children’s developmental outcomes. Adv Child Dev Behav.

[7] Skouteris H, Green R, Chung A, Bergmeier H, Amir LH, Baidwan SK (2022). Nurturing children’s development through healthy eating and active living: time for policies to support effective interventions in the context of responsive emotional support and early learning. Health Soc Care Community.

[8] McCarton L, O’Hogain S, Reid A, Leal Fo W (2020). Encyclopedia of the UN sustainable development goals.

[9] World Health Organization (2016). Global strategy for women’s, children’s and adolescents’ health (2016–2030).

[10] World Health Organization, United Nations Children’s Fund, World Bank Group (2018). Nurturing care for early childhood development: a framework for helping children survive and thrive to transform health and human potential.

[11] Zhang L, Ssewanyana D, Martin MC, Lye S, Moran G, Abubakar A (2021). Supporting child development through parenting interventions in low- to middle-income countries: an updated systematic review. Front Public Health.

[12] Brazelton TB, Greenspan SI (2002). As necessidades essenciais das crianças: o que toda criança precisa para crescer, aprender e se desenvolver.

[13] Hermenau K, Goessmann K, Rygaard NP, Landolt MA, Hecker T (2017). Fostering child development by improving care quality: a systematic review of the effectiveness of structural interventions and caregiver trainings in institutional care. Trauma Violence Abuse.

[14] Teixeira KP, Gabatz RI, Milbrath VM, Vaz EC, Klumb MM, Silva LL (2023). Professionals who exercise care in childhood residential institutions: an integrative review. Res Soc Dev.

[15] Aromataris E, Lockwood C, Porritt K, Pilla B, Jordan Z (2024). JBI manual for evidence synthesis.

[16] Tricco AC, Lillie E, Zarin W, O’Brien KK, Colquhoun H, Levac D (2018). PRISMA extension for scoping reviews (PRISMA-ScR): checklist and explanation. Ann Intern Med.

[17] Biblioteca Virtual em Saúde (2025). Descritores em Ciências da Saúde (DeCS).

[18] Sena MC, Silva FMF, Marques HR, Bastos PRHO (2020). Ativismo Judicial e a Implantação do Programa Família Acolhedora no Estado de Mato Grosso do Sul. Interações.

[19] Hoffmann TC, Glasziou PP, Boutron I, Milne R, Perera R, Moher D (2014). Better reporting of interventions: template for intervention description and replication (TIDieR) checklist and guide. BMJ.

[20] Fensterseifer JM (2021). Cuidadoras de crianças institucionalizadas: intervenção e cuidados [dissertação].

[21] McCall RB, Groark CJ, Hawk BN, Julian MM, Merz EC, Rosas JM (2019). Early caregiver-child interaction and children’s development: lessons from the St. Petersburg-USA orphanage intervention research project. Clin Child Fam Psychol Rev.

[22] Bakermans-Kranenburg MJ, van Ijzendoorn MH, Juffer F (2008). Earlier is better: a meta-analysis of 70 years of intervention improving cognitive development in institutionalized children. Monogr Soc Res Child Dev.

[23] Warner HA, McCall RB, Groark CJ, Kim KH, Muhamedrahimov RJ, Palmov OI (2017). Caregiver-child interaction, caregiver transitions, and group size as mediators between intervention condition and attachment and physical growth outcomes in institutionalized children. Infant Ment Health J.

[24] Lecannelier F, Silva JR, Hoffmann M, Melo R, Morales R (2014). Effects of an intervention to promote socioemotional development in terms of attachment security: a study in early institutionalization in Chile. Infant Ment Health J.

[25] Hawk BN, McCall RB, Groark CJ, Muhamedrahimov RJ, Palmov OI, Nikiforova NV (2018). Caregiver sensitivity and consistency and children’s prior family experience as contexts for early development within institutions. Infant Ment Health J.

[26] McCall RB, Groark CJ, Fish L, Muhamedrahimov RJ, Palmov OI, Nikiforova NV (2013). Maintaining a social-emotional intervention and its benefits for institutionalized children. Child Dev.

[27] Cavalheiro MG, Lopes-Herrera SA (2020). Estimulação de linguagem para cuidadores de crianças institucionalizadas: elaboração de um blog. Arch Health Invest.

[28] Julian MM, Li J, Wright A, Jimenez-Etcheverria PA, Tulviste T, Best D, Gibbons J (2019). Children’s social worlds in cultural context.

[29] McCall RB, Fish LA, Groark CJ, Muhamedrahimov RJ, Palmov O, Nikiforova NV (2012). The role of transitions to new age groups in the development of institutionalized children. Infant Ment Health J.

[30] Berument SK (2013). Environmental enrichment and caregiver training to support the development of birth to 6-year-olds in Turkish orphanages. Infant Ment Health J.

[31] Taneja V, Sriram S, Beri RS, Sreenivas V, Aggarwal R, Kaur R (2002). ‘Not by bread alone’: impact of a structured 90-minute play session on development of children in an orphanage. Child Care Health Dev.

[32] McCall RB, Groark CJ, Fish L, Harkins D, Serrano G, Gordon K (2010). A socioemotional intervention in a Latin American orphanage. Infant Ment Health J.

[33] Groark CJ, McCall RB, McCarthy SK, Eichner JC, Warner HA, Salaway J (2013). The effects of a social–emotional intervention on caregivers and children with disabilities in two Central American institutions. Infants Young Child.

[34] Wright AC, Lamsal D, Ksetree M, Sharma A, Jaffe K (2014). From maid to mother: transforming facilities, staff training, and caregiver dignity in an institutional facility for young children in Nepal. Infant Ment Health J.

[35] Hermenau K, Kaltenbach E, Mkinga G, Hecker T (2015). Improving care quality and preventing maltreatment in institutional care: a feasibility study with caregivers. Front Psychol.

[36] Ferrante C, Sorgato P, Fioravanti M, Pacione L, Arduino G, Ghersi S (2022). Supporting caregivers remotely during a pandemic: comparison of WHO Caregiver Skills Training delivered online versus in person in public health settings in Italy. J Autism Dev Disord.

[37] Valenstein-Mah H, Greer N, McKenzie L, Hansen LP, Strom TQ, Stirman SW (2020). Effectiveness of training methods for delivery of evidence-based psychotherapies: a systematic review. Implement Sci.

[38] Valenstein-Mah H, Greer N, McKenzie L, Hansen L, Strom TQ, Wiltsey Stirman S (2020). Effectiveness of training methods for delivery of evidence-based psychotherapies: a systematic review. Implement Sci.

[39] Day SD, Nguyen K-H, Comans T, Clemson L, Laver K (2021). Professional development training preferences of occupational therapists working with older adults in Australia: a discrete choice experiment. Aust Occup Ther J.

[40] López-de-la-Fuente MJ, Herrero P, García-Foncillas R, Gómez-Trullén EM (2021). Contextual, client-centred coaching following a workshop: assistants capacity building in special education. Int J Environ Res Public Health.

[41] Valanci-Aroesty S, Alhassan N, Feldman LS, Landry T, Mastropietro V, Fiore J (2020). Implementation and effectiveness of coaching for surgeons in practice: a mixed studies systematic review. J Surg Educ.

[42] Braithwaite J, Ludlow K, Testa L, Herkes J, Augustsson H, Lamprell G (2020). Built to last? The sustainability of healthcare system improvements, programmes and interventions: a systematic integrative review. BMJ Open.

[43] Hall A, Shoesmith A, Doherty E, McEvoy B, Mettert KD, Lewis C (2022). Evaluation of measures of sustainability and sustainability determinants for use in community, public health, and clinical settings: a systematic review. Implement Sci.

[44] Jeong J, Ahun MN, Gunaratna NS, Ambikapathi R, Mapendo F, Galvin L (2024). Effects of engaging fathers and bundling parenting and nutrition interventions on early child development and maternal and paternal parenting in Mara, Tanzania: a factorial cluster-randomized controlled trial. J Child Psychol Psychiatry.

[45] Ivanova K (2024). Review on multi-component training programs for parents of children with neurodevelopmental disabilities and direct-care staff. J Educ Res Educ.

[46] Neder K, Ferreira LDMP, Amorim KS (2020). Coconstrução do apego no primeiro semestre de vida: o papel do outro nessa constituição. Psicol USP.

[47] Sand H, Sticca F, Eichelberger DA, Wehrle FM, Simoni H, Jenni OG (2024). Raised in conditions of psychosocial deprivation: effects of infant institutionalization on early development. Child Youth Serv Rev.

[48] Altafim ERP, Souza M, Teixeira L, Brum D, Velho C (2023). O cuidado integral e a parentalidade positiva na primeira infância.

[49] Luz RMD, Marinho DCB, Lima APE, Coriolano-Marinus MWL (2022). Educational interventions in child development and health literacy assumptions: an integrative review. Rev Bras Enferm.

[50] Luoto JE, Lopez Garcia I, Aboud FE, Singla DR, Fernald LCH, Pitchik HO (2021). Group-based parenting interventions to promote child development in rural Kenya: a multi-arm, cluster-randomised community effectiveness trial. Lancet Glob Health.

[51] Dahlberg M, Nordmyr J, Gunnarsdottir H, Forsman AK (2023). The evidenced effects of early childhood interventions to promote mental health and parenting in the Nordic countries: a systematic review. Int J Ment Health Promot.

[52] Núcleo de Cultura de Paz e Inovação (2024). Caminhos e aprendizados para iniciativas focadas na primeira infância.

[53] Center on the Developing Child (2017). IDEAS impact framework components.

[54] Vicente JB, Pegorin TC, Santos ALO, Ramallo Veríssimo ML (2023). Intervenciones para el desarrollo infantil basadas en el Modelo Touchpoints: revisión de alcance. Rev Lat Am Enfermagem.

