# Supplementary material for: Interventions to promote child development in institutionalized children: a scoping review
Source: Rev Esc Enferm USP. 2025 Nov 17;59:e20250044. doi: 10.1590/1980-220X-REEUSP-2025-0044en (PMC12624873; doi:10.1590/1980-220X-REEUSP-2025-0044en)
Supplement: Translation

# Translation: Intervenções para promoção do desenvolvimento infantil de crianças institucionalizadas: revisão de escopo

**DOI:** 10.1590/1980-220X-REEUSP-2025-0044pt

**Authors:** Vanessa Martins, Vitória da Silva Porto, Katherine Solís-Cordero, Clariana Vitória Ramos de Oliveira, Jéssica Batistela Vicente, Daniela Doulavince Amador, Maria de La Ó Ramallo Veríssimo, Samara Macedo Cordeiro

**Affiliations:** 1 Universidade Estadual de Campinas, Faculdade de Enfermagem, Campinas, SP, Brasil.; 2 Universidad de Costa Rica, Escuela de Enfermería, San José, Costa Rica.; 3 University of Nevada Las Vegas, School of Nursing, Las Vegas, Nevada, United States.; 4 Universidade Federal do Paraná, Coordenadoria de Atenção Integral à Saúde Estudantil, Curitiba, PR, Brasil.; 5 Universidade de São Paulo, Escola de Enfermagem, São Paulo, SP, Brasil.

**Keywords:** Desenvolvimento Infantil, Criança Institucionalizada, Cuidadores

## Abstract

**Objetivo:**

Mapear as características e os resultados das intervenções realizadas com cuidadores de crianças institucionalizadas para promoção do desenvolvimento infantil.

**Método:**

Revisão de escopo segundo as diretrizes do JBI, realizada em oito bases de dados, literatura cinzenta e listas de referência dos estudos selecionados. A questão da revisão foi: quais são as características e os resultados das intervenções realizadas com cuidadores para promoção do desenvolvimento infantil de crianças de 0 a 6 anos institucionalizadas? Utilizou-se o *software* Rayyan^®^ para seleção dos estudos. A análise foi descritiva.

**Resultados:**

Foram incluídos 17 estudos, que relataram intervenções do tipo treinamento, associados ou não a mudanças no processo de trabalho e na organização estrutural da instituição, além de treinamento por meio da criação de ambiente virtual de aprendizagem. Houve efeitos positivos no desenvolvimento infantil, mas a heterogeneidade das intervenções e resultados não permitiu verificar quais são seus aspectos mais relevantes. Os principais conteúdos abordados foram os cuidados responsivos.

**Conclusão:**

A diversidade de modelos de treinamento profissional não permite afirmar quais aspectos são mais relevantes na formação dos cuidadores, demandando estudos mais robustos sobre o tema.

## INTRODUÇÃO

A primeira infância, de 0 aos 6 anos^(1)^, é um período marcado por vulnerabilidades e oportunidades^(2)^, no qual a criança passa por mudanças físicas, cognitivas e psicossociais, e o cérebro se desenvolve de forma acelerada, sendo, portanto, um momento estratégico para promoção de seu desenvolvimento. Muitas crianças podem passar por essa fase enquanto estão temporariamente separadas de suas famílias e/ou cuidadores primários em um local de acolhimento institucional até que possam ser reunidas a eles ou encaminhadas para famílias substitutas^(3)^. Segundo o Conselho Nacional de Justiça, cerca de 34 mil crianças vivem em situação de acolhimento no Brasil^(4)^.

O desenvolvimento infantil (DI) é um processo ativo e único para cada criança, expresso por continuidade e mudanças na aquisição de habilidades motoras, cognitivas, psicossociais e de linguagem, e os primeiros anos de vida são o alicerce deste processo^(5)^. Um cuidado voltado às necessidades de desenvolvimento possibilita à criança alcançar todo o seu potencial^(6)^.

Promover o desenvolvimento das crianças é uma prioridade a nível nacional e internacional^(7)^. No Brasil, o Marco Legal da Primeira Infância^(1)^ estabelece como prioridade a criação de programas e políticas públicas que foquem no desenvolvimento integral da primeira infância, além de ressaltar a importância da qualificação dos profissionais, que desempenham um papel fundamental ao fornecer assistência direta ou apoiar outros cuidadores, empregando técnicas pedagógicas participativas que estimulem a reflexão e decisão por comportamentos apropriados às necessidades individuais de cada criança.

Mundialmente, a importância desta temática foi endossada pelos Objetivos de Desenvolvimento Sustentável (ODS)^(8)^, pela Estratégia Global para a Saúde das Mulheres, Crianças e Adolescentes (2016–2030)^(9)^ e pelo *Nurturing Care*, que prioriza as intervenções para promoção do desenvolvimento integral na primeira infância, incluindo o fortalecimento das competências parentais para o cuidado responsivo às necessidades essenciais da criança^(10)^.

A partir do conhecimento construído, que demonstra que as interações na primeira infância afetam os circuitos cerebrais e favorecem o desenvolvimento emocional e intelectual^(2)^, a promoção do desenvolvimento infantil, e a proteção e o cuidado das crianças deixaram de ser vistos como responsabilidade exclusiva de seus pais e familiares, passando a ser foco de amplo compromisso político e social. No contexto de crianças em instituições de acolhimento, esse compromisso se torna ainda mais essencial, uma vez que as crianças acolhidas vivenciam experiências potencialmente adversas, muitas vezes envolvendo violência e possível estresse tóxico.

As intervenções destinadas ao desenvolvimento infantil têm promovido uma ampliação do conhecimento no campo da ciência do desenvolvimento. Entre as intervenções, evidências indicam que o cuidado responsivo, especialmente envolvendo métodos de promoção da interação entre o cuidador-criança, é o mais efetivo para o desenvolvimento infantil^(10,11)^. O cuidado responsivo possibilita atender às necessidades essenciais das crianças, que envolvem o estabelecimento de relacionamentos saudáveis e permanentes, segurança física, respeito às características individuais, experiências compatíveis com o momento do desenvolvimento, e convívio em uma comunidade estável e amparadora^(12)^.

Revisão sistemática^(13)^ identificou que intervenções estruturais realizadas em abrigo e ações formativas com cuidadoras foram efetivas para promoção do desenvolvimento infantil. Entretanto, apesar de evidências indicarem a efetividade dessas intervenções, a literatura ainda carece de uma descrição detalhada de suas características, especialmente no contexto brasileiro. Esta lacuna dificulta a replicação, adaptação e escalonamento de práticas baseadas em evidências, comprometendo a qualidade do cuidado oferecido.

Ao considerar que o cuidado nas instituições de abrigamento infantil é realizado por cuidadores leigos, cuja formação mínima requerida é o ensino médio^(14)^, capacitações sobre o crescimento e o desenvolvimento infantil se tornam necessárias.

A qualificação do cuidado e das relações entre crianças e seus cuidadores pode ser potencializada por intervenções educativas. Isso justifica conhecer e selecionar evidências para elaborar um programa de promoção do desenvolvimento infantil no contexto da institucionalização de crianças menores de 6 anos. Portanto, este estudo teve como objetivo mapear as características e os resultados de intervenções realizadas com cuidadores de crianças institucionalizadas para promoção do desenvolvimento infantil.

Não foram encontrados estudos que permitam a compreensão das características das intervenções realizadas com cuidadoras de crianças institucionalizadas para promoção do desenvolvimento infantil. A realização de análise detalhada e abrangente dessas intervenções torna possível a identificação de características que favoreçam sua implementação, replicação ou desenvolvimento de novos estudos fundamentados nos achados identificados.

## MÉTODO

### Tipo de Estudo

Trata-se de uma revisão de escopo, método que mapeia e caracteriza a extensão das evidências sobre um campo de pesquisa, podendo evidenciar as lacunas do conhecimento e demonstrar a relevância e necessidade de estudo mais aprofundados sobre determinadas áreas^(15)^. Este estudo foi desenvolvido e estruturado segundo as recomendações da JBI^(15)^, conforme detalhado nos tópicos a seguir, e o *checklist Preferred Reporting Items for Systematic reviews and Meta-Analyses extension for Scoping Reviews* (PRISMA-ScR)^(16)^. O protocolo da revisão foi registrado na plataforma *Open Science Framework* (https://doi.org/10.17605/OSF.IO/HN8E2).

As etapas metodológicas do estudo foram: formulação da questão da pesquisa, utilizando o mnemônico População, Conceito e Contexto (PCC); definição de critérios de inclusão e exclusão; definição dos tipos de estudos e fontes; elaboração de estratégia de busca; identificação das bases de dados; busca e seleção de estudos; extração e análise dos dados; e construção do relatório^(15)^.

### Identificação da Questão de Pesquisa

A elaboração da questão norteadora foi baseada na estrutura mnemônica PCC (População, Conceito e Contexto), sendo População (P): cuidadores; Conceito ©: intervenções para promoção do desenvolvimento infantil; Contexto ©: institucionalização infantil. Com esta combinação mnemônica, definiu-se a seguinte questão norteadora: quais são as características e os resultados das intervenções realizadas com cuidadores para promoção do desenvolvimento infantil de crianças de 0 a 6 anos institucionalizadas?

Foram consideradas crianças institucionalizadas aquelas que estão recebendo serviços de longa duração, ou que residem em um ambiente institucional^(17)^, e intervenções para promoção do desenvolvimento infantil, ações que promovem o desenvolvimento físico, socioemocional, cognitivo e motor, implementadas para crianças de 0 a 6 anos de idade. A escolha da faixa etária de 0 a 6 anos ocorreu porque esse período corresponde à primeira infância, uma fase importante para o desenvolvimento físico, emocional, mental e social da criança, sendo, portanto, estratégico para a promoção do seu desenvolvimento infantil.

### Critérios de Seleção

Foram incluídos artigos científicos primários e revisões, descritivos ou analíticos, teses e dissertações que responderam à pergunta da revisão. Não houve limitação temporal, de localidades geográficas ou de idiomas.

Foram excluídos os estudos que não contemplaram a população, o conceito e o contexto de interesse, ou que não reportaram a aplicação de uma intervenção, bem como editoriais, cartas ao editor e artigos de opinião. Além disso, também foram excluídos os estudos em que a criança estava sob a responsabilidade de uma família acolhedora, programa criado em 2009 cujo objetivo é enviar as crianças às famílias selecionadas, capacitadas e acompanhadas pelo poder público municipal^(18)^, visto que, nesses casos, a criança não estava em ambiente institucional. Os documentos duplicados foram considerados apenas uma vez.

### Estratégias de Busca

A busca eletrônica foi realizada entre setembro e dezembro de 2023, e atualizada em abril de 2025 com o auxílio de uma bibliotecária utilizando os Descritores em Ciências da Saúde (DeCS) e *Medical Subject Headings* (MeSH), em conjunto com os operadores booleanos *AND* e *OR* nas bases de dados e na literatura cinzenta (Quadro 1). Também foi realizada uma busca manual na lista de referências dos artigos incluídos.

**Quadro 1.** Estratégias de busca segundo bases de dados e literatura cinzenta – São Paulo, SP, Brasil, 2025.

Base de dados e literatura cinzenta	Estratégia de busca
PubMed	(((Child Development[MeSH Terms]) OR (“Child Development”[Title/Abstract] OR “Infant Development”[Title/Abstract])) AND ((Child, Institutionalized[MeSH Terms]) OR (“Child, Institutionalized”[Title/Abstract] OR “Institutionalized Child”[Title/Abstract] OR “Institutionalized Children”[Title/Abstract]))) AND ((Caregivers[MeSH Terms]) OR (Caregivers[Title/Abstract] OR Caregiver[Title/Abstract] OR Carers[Title/Abstract] OR Carer[Title/Abstract] OR “Care Givers”[Title/Abstract] OR “Care Giver”[Title/Abstract] OR “Spouse Caregivers”[Title/Abstract] OR “Spouse Caregiver”[Title/Abstract] OR “Informal Caregivers”[Title/Abstract] OR “Informal Caregiver”[Title/Abstract]))
Biblioteca Virtual em Saúde	(“Child Development” OR “Infant Development”) AND (“Child, Institutionalized” OR “Institutionalized Child” OR “Institutionalized Children”) AND (caregivers OR caregiver OR carers OR carer OR “Care Givers” OR “Care Giver” OR “Spouse Caregivers” OR “Spouse Caregiver” OR “Informal Caregivers” OR “Informal Caregiver”)
Scopus	(TITLE-ABS-KEY (“Child Development” OR “Infant Development”)) AND (TITLE-ABS-KEY (“Child, Institutionalized” OR “Institutionalized Child” OR “Institutionalized Children”)) AND (TITLE-ABS-KEY (caregivers OR caregiver OR carers OR carer OR “Care Givers” OR “Care Giver” OR “Spouse Caregivers” OR “Spouse Caregiver” OR “Informal Caregivers” OR “Informal Caregiver”))
Scientific Electronic Library Online	“Child Development” AND “Child, Institutionalized” AND “caregivers”
Cochrane	“Child Development” AND “Child, Institutionalized” AND “caregivers”
Portal de Periódicos da Coordenação de Aperfeiçoamento de Pessoal de Nível Superior	“Child Development” AND “Child, Institutionalized” AND “caregivers”
Web of Science	“Child Development” AND “Child, Institutionalized” AND “caregivers”
PsycINFO	“Child Development” AND “Child, Institutionalized” AND “caregivers”
Google Scholar	“Child Development” AND “Child, Institutionalized” AND “caregivers”
Biblioteca Digital Brasileira de Teses e Dissertações	“Child Development” AND “Child, Institutionalized” AND “caregivers”

### Seleção dos Estudos

Os resultados provenientes de todas as plataformas de busca foram transferidos para o Rayyan® (*software* empregado na seleção de estudos em métodos de síntese do conhecimento) para eliminar estudos duplicados e efetuar a seleção das pesquisas. A seleção ocorreu em duas fases. A primeira fase foi realizada por dois revisores independentes treinados que efetuaram a leitura e avaliação dos títulos e resumos dos registros identificados, com a pré-seleção dos estudos potencialmente elegíveis. Nos casos de discordância, foi convidado um terceiro revisor para decidir sobre a inclusão ou não dos artigos. O terceiro revisor era experiente na temática do desenvolvimento infantil e na realização de revisões sistemáticas de literatura. Na segunda fase, conduzida por uma das revisoras, foi realizada a avaliação do texto na íntegra dos estudos pré-selecionados para confirmação de sua elegibilidade.

### Extração e Síntese dos Dados

Após a definição da amostra de estudos incluídos, extraíram-se os dados em formulário específico para identificação do estudo (título, autor, país, ano de publicação, tipo de estudo e objetivo). Os dados extraídos para a caracterização das intervenções foram população, tipo de intervenção, contexto de realização das intervenções e domínio do desenvolvimento, acrescidas das recomendadas pelo *Template for Intervention Description and Replication* (*TIDieR*)^(19)^, como referencial teórico utilizado, profissional responsável pela intervenção, conteúdo da intervenção, materiais e estratégias utilizadas, modo de entrega, dose (número de vezes, duração e período), e resultados da intervenção. O *TIDieR* objetiva melhorar a descrição detalhada das intervenções para permitir sua replicabilidade.

Essas informações foram inseridas em planilha eletrônica do *Microsoft Office World* ^®^ e permitiram a síntese e interpretação dos estudos.

### Aspectos Éticos

Por se tratar de pesquisa de revisão, dispensou-se apreciação do Comitê de Ética e Pesquisa para seu desenvolvimento.

## RESULTADOS

Um total de 417 estudos foram inicialmente identificados, sendo 274 duplicatas excluídas por meio da plataforma Rayyan®, resultando em 143 estudos únicos. Entre esses, 115 foram excluídos após avaliação dos títulos e resumos, e 28 foram submetidos à leitura na íntegra. Na fase de leitura completa, 17 artigos foram excluídos por não abordarem as características e os resultados das intervenções realizadas com cuidadores para promoção do desenvolvimento infantil de crianças de 0 a 6 anos institucionalizadas, e 11 estudos foram incluídos na pesquisa. Pela busca manual na lista de referências, foram identificados seis novos estudos, totalizando uma amostra final com 17 estudos (Figura 1).

**Figura 1:** Fluxograma do processo de seleção dos artigos para a revisão de escopo adaptado do *PRISMA-ScR 2020 flow diagram for new systematic reviews which included searches of databases, registers and other sources*. São Paulo, SP, Brasil, 2025.

Quanto ao tipo de estudo, conforme descrito nos artigos, identificaram-se estudos qualitativos^(20)^, revisões de literatura^(13,21,22)^, estudos de intervenção quase-experimental^(23,24)^, estudos de coorte^(25,26)^, estudos metodológicos^(27)^, capítulos de livro^(28)^, estudos de intervenção não randomizados^(29,30)^, estudos longitudinais prospectivos^(31)^, intervenções piloto^(32,33)^, estudos de caso^(34)^ e estudos de viabilidade^(35)^. As pesquisas foram publicadas entre 2002 e 2021, e realizadas no Brasil^(20,27)^, nos Estados Unidos^(13,21,22,28,32)^, na Rússia^(23,25,26,29)^, no Chile^(24)^, na Índia^(31)^, na Turquia^(30)^, no Nepal^(34)^, na Tanzânia^(35)^ e nos países da América Central^(33)^.

No Quadro 2, são apresentados os objetivos e resultados das intervenções.

**Quadro 2.** Identificação dos estudos segundo autor, país, ano de publicação, objetivo do estudo e resultados – São Paulo, SP, Brasil, 2025.

Título	Autor País Ano	Objetivo	Resultado
Cuidadoras de crianças institucionalizadas: intervenção e cuidados	Jacqueline Müllich Fensterseifer Brasil 2021	Apresentar os conhecimentos produzidos sobre a forma como são realizados os cuidados aos bebês em situação de acolhimento.	As intervenções auxiliaram na promoção de vínculos afetivos de melhor qualidade entre cuidadores e crianças. Houve melhorias na saúde mental e no bem-estar dos cuidadores e das crianças.
Early Caregiver–Child Interaction and Children’s Development: Lessons from the St. Petersburg-USA Orphanage Intervention Research Project	Robert B McCall *et al*. EUA 2018	Realizar revisão dos estudos que tratam do desenvolvimento das crianças que residem em instituições na Federação Russa ou colocadas com famílias nos EUA e na Rússia.	A melhora das interações entre cuidadores e crianças em instituições impacta positivamente o desenvolvimento físico e comportamental infantil. As cuidadoras melhoraram sua resposta de cuidado diante do choro das crianças.
Caregiver-child Interaction, Caregiver Transitions, And Group Size As Mediators Between Intervention Condition And Attachment And Physical Growth Outcomes In Institutionalized Children.	Hilary A Warner *et al*. Rússia 2017	Descrever análise secundária de dados do estudo de intervenção russo para determinar se a qualidade da interação cuidador-criança, as transições entre cuidadores e o tamanho do grupo influenciam o efeito da intervenção no apego e no crescimento físico das crianças.	Houve melhora da qualidade de interações entre as cuidadoras e crianças. Crianças que viviam nas instituições submetidas à intervenção obtiveram altura, peso e perímetro cefálico significativamente maiores do que as crianças que não passaram por intervenção.
Effects of an intervention to promote socioemotional development in terms of attachment security: a study in early institutionalization in Chile	Felipe Lecannelier *et al*. Chile 2014	Avaliar o estado psicoafetivo de bebês em condições institucionais nas áreas de apego, desenvolvimento psicomotor, temperamento e desenvolvimento social, além de analisar a efetividade de um programa de promoção do desenvolvimento socioemocional implementado nos centros residenciais, considerando a segurança do apego como variável organizadora.	Houve melhora na relação emocional entre a criança e o cuidador, na capacidade de atenção e na segurança do apego, com um aumento da porcentagem de bebês com apego seguro.
Fostering Child Development by Improving Care Quality: A Systematic Review of the Effectiveness of Structural Interventions and Caregiver Trainings in Institutional Care	Katharin Hermenau *et al*. EUA 2016	Revisar sistematicamente as evidências atuais sobre a eficácia das intervenções estruturais e da formação de cuidadores em cuidados institucionais, a fim de promover o desenvolvimento das crianças por meio da melhoria da qualidade dos cuidados e da prevenção de maus-tratos.	A qualidade do cuidado infantil, especialmente nos primeiros anos de vida, tem impacto crucial no desenvolvimento e funcionamento mental saudável em períodos posteriores. Houve efeitos benéficos sobre o desenvolvimento emocional, social e cognitivo das crianças em ambiente institucional.
Caregiver sensitivity and consistency and children’s prior family experience as contexts for early development within institutions	Brandi N Hawk *et al*. Rússia 2018	Examinar a associação entre sensibilidade do cuidador e consistência com o desenvolvimento socioemocional e cognitivo de crianças pequenas institucionalizadas.	Melhorar a sensibilidade e consistência dos cuidadores pode ter impacto positivo no desenvolvimento cognitivo e socioemocional das crianças institucionalizadas, especialmente no primeiro ano.
Maintaining a Social-Emotional Intervention and its Benefits for Institutionalized Children	Robert B McCall *et al*. Rússia 2013	Relatar a manutenção de uma intervenção socioemocional realizada em orfanatos russos utilizando a equipe de cuidadores regulares.	A intervenção de treinamento e mudança estrutural mostrou melhorias no desenvolvimento durante e após a intervenção; porém, a rotatividade de pessoal pode prejudicar sua continuidade e afetar a consistência dos cuidadores, impactando os resultados de desenvolvimento das crianças.
Estimulação de linguagem para cuidadores de crianças institucionalizadas: elaboração de um blog	Maria Gabriela Cavalheiro; Simone Aparecida Lopes-Herrera Brasil 2019	Elaborar um ambiente virtual de aprendizagem na área de estimulação de linguagem voltado à capacitação de cuidadores de crianças institucionalizadas em abrigos, em um contexto de Educação a Distância.	Os resultados destacaram o potencial ações de Educação a Distância na capacitação de profissionais ligados à população de risco para alterações de linguagem.
Earlier is better: a meta-analysis of 70 years of intervention improving cognitive development in institutionalized children	Marian J. Bakermans-Kranenburg, Marinus H. Van IJzendoorn, Mulher Juffer EUA 2008	Revisar estudos de intervenção voltados à melhoria do desenvolvimento de crianças institucionalizadas.	Intervenções realizadas em instituições demonstram melhorias significativas no desenvolvimento cognitivo, da linguagem e socioemocional das crianças. Além disso, algumas intervenções também beneficiaram a saúde física, o crescimento e o desenvolvimento motor delas.
Young Children in Institutional Care: Characteristics of Institutions, Children’s Development, and Interventions in Institutions	Megan M Julian *et al*. EUA 2019	Descrever os ambientes sociais de instituições ao redor do mundo e intervenções realizadas nessas localidades.	Aprimorar a qualidade dos cuidados em instituições pode ter impactos significativos no desenvolvimento das crianças que lá residem, especialmente quando os cuidadores interagem regularmente com as mesmas crianças, aumentando, assim, sua sensibilidade e capacidade de resposta durante as atividades diárias.
The role of transitions to new age groups in the development of institutionalized children	Robert B McCall *et al*. Rússia 2012	Descrever os efeitos da transição para novos grupos etários e cuidadores no desenvolvimento geral de crianças institucionalizadas.	A rotatividade de quartos, cuidadores e grupos pode ser prejudicial para crianças institucionalizadas, mas é mitigada pela interação sensível entre cuidador-criança. O treinamento para melhorar essa interação e evitar transições levou a um desenvolvimento mais positivo e menos estressante.
‘Not by bread alone’: impact of a structured 90-minute play session on development of children in an orphanage	V. Taneja *et al*. Índia 2002	Desenvolver um programa de intervenção de jogo estruturado.	As crianças apresentaram maior responsividade, tiveram melhor controle da cabeça e do corpo, e mostraram-se mais ativas. As cuidadoras se apresentavam relutantes no início da intervenção quanto ao aumento da carga de trabalho, porém, no final, ficaram entusiasmadas.
A socioemotional intervention in a Latin American orphanage	Robert B McCall *et al*. EUA 2012	Avaliar como uma intervenção socioemocional que enfatizou treinamento e assistência técnica poderia afetar o desenvolvimento das crianças institucionalizadas.	Após a intervenção, houve uma melhoria significativa nas interações entre cuidadores e crianças, uma abordagem mais calorosa, atenciosa, sensível e envolvente, especialmente para crianças mais jovens. As melhorias foram mais pronunciadas durante atividades como alimentação, banho/troca de roupa e brincadeiras livres.
Environmental Enrichment and Caregiver Training to Support the Development of Birth to 6-Year-Olds in Turkish Orphanages	Sibel Kazak Berument Turquia 2013	Desenvolver um programa de intervenção que integra enriquecimento ambiental e capacitação de cuidadores, visando aprimorar a linguagem e o desenvolvimento cognitivo de bebês e crianças pequenas que residem em ambientes institucionais na Turquia.	O programa de intervenção foi eficaz no aumento da qualidade do atendimento nos orfanatos, também tendo efeito no desenvolvimento linguístico e cognitivo dos bebês e pré-escolares neste estudo.
From maid to mother: transforming facilities, staff training, and caregiver dignity in an institutional facility for young children in Nepal	Amy Conley Wright *et al*. Nepal 2014	Relatar o caso de melhora da saúde, da segurança e do desenvolvimento das crianças de 0 a 6 anos de idade em um orfanato no Nepal.	Houve a diminuição de doenças infecciosas e um aumento nas interações sociais entre as crianças e os cuidadores. Além disso, houve um progresso no desenvolvimento cognitivo e emocional das crianças após a implementação das intervenções, juntamente com uma melhoria na autoestima e na sensação de “dignidade” entre os cuidadores.
Improving care quality and preventing maltreatment in institutional care – a feasibility study with caregivers	Katharin Hermenau *et al*. Tanzânia 2015	Testar a viabilidade de uma intervenção para promoção do desenvolvimento infantil e avaliar mudanças nos índices de maus-tratos infantis.	A formação é viável mesmo em circunstâncias desafiadoras. O conteúdo abordado é aplicável no processo de trabalho do cuidador. As crianças também relataram melhoria no cuidado.
The Effects of a Social–Emotional Intervention on Caregivers and Children With Disabilities in Two Central American Institutions	Christina J Groark *et al*. América Central 2013	Relatar uma intervenção de projeto piloto para melhorar duas instituições centro-americanas que atendiam crianças com deficiências graves e múltiplas usando uma abordagem de treinamento de instrutores para encorajar cuidadores típicos da equipe a fornecer interações sensíveis, responsivas e respeitosas com as crianças.	Intervenção que prioriza o cuidado sensível e responsivo, juntamente com algumas técnicas básicas especializadas de posicionamento e manuseio, pode ser aplicada com êxito para aprimorar o desenvolvimento físico e comportamental/mental de crianças institucionalizadas com deficiências graves e múltiplas.

Legenda: EUA – Estados Unidos da América.

Todos os estudos foram realizados com cuidadoras de crianças institucionalizadas, em sua maioria na própria instituição de acolhimento, e em um dos estudos, a intervenção do tipo treinamento foi realizada para as cuidadoras em escola primária da região^(35)^. A intervenção foi realizada de forma virtual em um estudo^(27)^, em treze presencialmente^(13,20,21,22,23,24,25,26,29,30,31,33,35)^, e em três estudos, não há descrição dessa informação^(28,29,34)^. Entre as intervenções presenciais, cinco foram realizadas em grupos^(20,24,29,33,35)^, e oito^(13,21,22,23,25,26,28,30)^ não explicitam se a intervenção foi feita em grupo ou de forma individual. Dois estudos citam que, além de realizarem um treinamento em grupo, foi realizada uma supervisão individual dos cuidadores após o treinamento^(25,33)^.

Quanto ao uso de referencial teórico para construção da intervenção: um estudo^(20)^ utilizou documentos das áreas da saúde, psicologia social, psicanálise e assistência social, o Estatuto da Criança e do Adolescente, e as Orientações Técnicas para Serviços de Acolhimento de Crianças e Adolescentes de 2009; três^(24,25,29)^ utilizaram a Teoria do Apego de John Bowlby; um^(31)^ utilizou a Teoria Social da Aprendizagem; um^(32)^ utilizou um programa já conhecido para cuidados infantis em associação com preceitos do Instituto Pikler de Budapeste; um^(35)^ seguiu as diretrizes da Academia Americana de Pediatria (1999) e conceitos de treinamento pré-estabelecidos; e dez estudos^(13,22,23,25,26,27,28,30,33,34)^ não mencionaram referencial teórico na elaboração da intervenção.

Os tipos de intervenção relatados foram: treinamentos das cuidadoras quanto a ações promotoras do desenvolvimento infantil durante o cuidado^(13,20,24,33)^; treinamento associado a mudanças no processo de trabalho e na organização estrutural da instituição^(21,23,25,26,28,29,30,32,34)^; e treinamento por meio da criação de um ambiente virtual de aprendizagem^(27)^. Um dos estudos utilizou uma técnica de modelagem, ou imitação, na qual os cuidadores eram incentivados a repetir as ações dos agentes da intervenção^(31)^, e um dos estudos, além do treinamento e mudanças estruturais, ofereceu uma assistência técnica de acompanhamento das cuidadoras^(32)^.

As intervenções tiveram como foco os domínios do desenvolvimento socioemocional^(13,20,21,22,23,24,25,26,28,29,32,34,35)^, motor^(21,23,26,31,32)^, cognitivo^(13,21,22,23,26,30,31,32,33,34)^, linguagem^(20,22,27,30,31,32)^ e físico^(33,34)^.

Os materiais utilizados para a intervenção variaram entre cartas e objetos do dia a dia de uma criança^(20)^, brinquedos e materiais de apoio, como trocadores e camas^(33)^, um manual de sensibilidade no apego^(24)^, brinquedos, livros e músicas pertinentes a cada faixa etária^(13,26,30,31,32)^, registro eletrônico na *web* com conteúdos teórico-práticos^(27)^, livros, e DVDs^(29)^. Sete estudos não descreveram os materiais utilizados na intervenção^(21,22,23,25,28,34,35)^.

Os profissionais responsáveis pela intervenção foram psicólogos^(20,31)^, psicólogos com apoio de enfermeiros^(35)^ e equipe multidisciplinar, com a presença de enfermeiros^(21,25)^ e fonoaudiólogos^(27)^. Em um dos estudos, os responsáveis foram profissionais de duas empresas com foco em desenvolvimento infantil, sendo uma delas sem fins lucrativos, além de receberem apoio de uma assistente social e uma psicóloga^(29)^. Sete estudos não especificam o profissional responsável pela intervenção^(13,22,23,24,26,28,29)^. Um estudo relata apenas que o profissional recebeu um treinamento prévio de pós-graduação para implementação da intervenção^(33)^.

Quanto à dose, os estudos variaram na distribuição de suas intervenções. Em quatro estudos, não há a descrição do período de realização, a duração e a frequência^(23,26,28,29)^. Uma pesquisa realizou a intervenção em 90 horas de observação e oficinas realizadas em quatro encontros^(20)^. Outras intervenções variaram de 12 e 14 encontros^(21)^; treinamento foi realizado em quatro horas^(24)^; houve treinamentos com nove módulos e 11 sessões de três horas para cada módulo^(25)^; houve um ano de treinamento semanal com duração de três horas cada sessão^(34)^; houve treinamento por duas semanas, oito horas por dia e seis dias por semana^(35)^; e houve nove sessões mensais de oito horas, complementadas com mini sessões mensais de uma a três horas^(33)^. Uma intervenção foi dividida em duas fases, sendo que a primeira ocorreu em um mês e a segunda, em dois meses, e cada sessão contida nessas fases ocorria em 90 minutos^(31)^. Outra intervenção ocorreu semanalmente no período de um ano^(32)^. Uma intervenção foi dividida em treinamento que ocorreu em 17 semanas com reuniões quinzenais com duração de 90 minutos cada e uma fase de supervisão de atividades, na qual oito encontros foram planejados, porém apenas dois foram executados^(30)^.

Os principais conteúdos trabalhados nas intervenções foram a aplicação de cuidados amorosos, sensíveis e responsivos^(21,23,25,26,28,29,30,32,33)^, formação de vínculo seguro entre bebês e seus cuidadores^(24,29,30)^, prevenção de maus-tratos^(35)^, aquisição de linguagem infantil, e a importância do cuidador nesse processo^(27)^. Além disso, também foi abordada a estimulação de crianças pré-escolares com temas de aprendizagem como cores, números e estações do ano^(30)^. Outro foco da intervenção foi o aprimoramento do trabalho diário e a melhoria das habilidades no momento do cuidado^(34)^.

As mudanças no processo de trabalho incluíram a redução do número das cuidadoras por grupo de crianças, redução do número de crianças por grupo para o cuidado, aumento da consistência de cuidados de uma cuidadora primária e criação da “hora da família”, um tempo de qualidade sem distrações e interferências externas entre as cuidadoras e crianças^(21,23,26,28,29)^. Um dos estudos proveu melhoria do cardápio, na higiene dos materiais e saneamento, e ainda instituiu visitas regulares de médicos e enfermeiros para cuidados de saúde^(34)^.

## DISCUSSÃO

Esta revisão de escopo evidenciou que intervenções direcionadas ao treinamento dos cuidadores de crianças institucionalizadas têm o potencial de promover melhorias significativas nos domínios socioemocional, motor, cognitivo, linguístico e físico das crianças envolvidas no contexto institucional.

A utilização de diferentes tipos de intervenções pode levar a resultados de implementação mais ou menos bem-sucedidos. Estudos mostram que alguns métodos de treinamento, como *workshops* presenciais sem acompanhamento, não aumentam a aquisição de habilidades do prestador de cuidados^(36,37)^, enquanto que métodos de formação mais intensivos podem levar a ganhos significativos nas competências dos prestadores de serviços.

Estudo que avaliou o treinamento de terapeutas em habilidades psicossociais encontrou evidências de que *workshops* com acompanhamento adicional e treinamentos de “multicomponentes” que incluíam observação, *feedback* e *coaching* aumentaram a aquisição de habilidades^(36)^. No entanto, ainda não está claro se métodos de formação mais rigorosos que podem melhorar os resultados dos prestadores de cuidados também melhoram os resultados da população cuidada^(38)^.

Estudo realizado na Austrália com terapeutas ocupacionais identificou a preferência por um suporte individual pós-treinamento para implementar o programa ensinado na prática, confirmando dados da literatura que apontam que o fornecimento de novas informações por si só tem um impacto limitado na mudança de comportamento^(39)^.

Outro estudo, conduzido com assistentes escolares, demonstrou que a combinação de uma abordagem contextual e *coaching* individual após um *workshop* é mais eficaz do que a realização de apenas um *workshop* para aprimorar o desempenho desses profissionais^(40)^. O *coaching* é definido como uma interação de aprendizagem onde um especialista observa o desempenho de outro indivíduo, oferecendo *feedback* objetivo e formativo visando promover a autorreflexão sobre o desempenho para alcançar objetivos previamente estabelecidos^(41)^.

Estudos demonstram que o monitoramento das atividades e comportamentos do cuidador, além do incentivo à adoção de práticas qualificadas, é crucial para a sustentabilidade da intervenção e das mudanças na promoção do desenvolvimento infantil^(32)^.

A sustentabilidade refere-se à continuidade dos efeitos positivos das intervenções e manutenção dos seus efeitos de longo prazo^(42)^. De acordo com a literatura, outros fatores que podem ter influência na sustentabilidade se referem à adaptação, caracterizada pela flexibilidade para transformações ao longo do tempo conforme o contexto, e à aprendizagem organizacional, que envolve formação contínua e baixa rotatividade da equipe. Além disso, o apoio organizacional e as parcerias são essenciais para criar um ambiente interno favorável. A integração com a missão institucional e a presença de “defensores” que possam sustentar as intervenções em períodos vulneráveis também são cruciais. Ademais, o financiamento deve ser contínuo e adequado, proveniente de diversas fontes, e a manutenção dos benefícios depende da percepção dos participantes de que a intervenção está atingindo os resultados inicialmente desejados^(42)^. Em revisão sistemática da literatura, tem-se que intervenções que incluem componentes de capacitação (tanto profissional quanto não profissional) tendem a ser mais sustentáveis do que aquelas que não incorporam tais atividades. Os indivíduos capacitados têm a capacidade de continuar proporcionando benefícios ao treinar membros da comunidade, estabelecendo uma rede de apoio ao programa^(43)^.

Entre as intervenções analisadas, destaca-se a efetivação de treinamentos com as cuidadoras associado a mudanças estruturais, e os resultados apontam uma melhoria na qualidade das interações cuidador-criança nessas instituições, melhores taxas de desenvolvimento infantil e sensibilização referente à importância de cuidados responsivos. Essas descobertas destacam efeitos positivos das intervenções que combinam treinamento com mudanças estruturais, fornecendo uma base sólida para futuras iniciativas voltadas para promoção do desenvolvimento infantil.

Além disso, esses estudos ressaltam a necessidade contínua de investimento em programas de treinamento para cuidadores, visando melhorar a qualidade dos cuidados prestados às crianças^(44)^. Pesquisa conduzida na Tanzânia com crianças menores de 18 meses de idade constatou que intervenções que incentivam o treinamento e a participação ativa de cuidadores e crianças, visando o desenvolvimento infantil, tiveram um impacto significativo no desenvolvimento cognitivo e linguístico^(44)^. A evidência de que intervenções que combinam treinamento com mudanças estruturais resulta em melhorias significativas nas interações cuidador-criança e no desenvolvimento infantil destaca a importância de abordagens integradas e intervenções com multicomponentes na promoção do bem-estar infantil^(44,45)^. No entanto, é crucial reconhecer que o sucesso dessas intervenções depende não apenas da implementação eficaz, mas também do apoio contínuo e do envolvimento das comunidades e instituições envolvidas. Portanto, futuras pesquisas e políticas devem se concentrar em garantir a sustentabilidade desses programas, bem como em adaptá-los às necessidades específicas de diferentes contextos culturais e sociais.

Ao elaborar as intervenções com as cuidadoras, entre aqueles estudos que apresentaram um referencial teórico, a teoria mais utilizada foi a Teoria do Apego de John Bowlby. Para Bowlby, o apego é considerado um mecanismo inato para os seres humanos, sendo o indivíduo biologicamente programado para reconhecer a presença de uma figura disponível que ofereça uma relação de trocas e proporcione um sentimento de segurança^(46)^. Usualmente, a figura do apego para a criança é a mãe biológica, entretanto, no caso de crianças institucionalizadas, há quebra desse paradigma, uma vez que ocorre o rompimento momentâneo ou contínuo dos laços familiares e, assim, as cuidadoras passam a exercer esse papel^(46)^.

As ferramentas necessárias para que as crianças atinjam seu potencial intelectual, social, emocional e físico são fornecidas por meio da atenção às necessidades essenciais^(12)^. A primeira necessidade a ser atendida é a de relacionamentos contínuos e sustentadores, em que a presença de uma pessoa principal no cuidado da criança, ou seja, um adulto de referência, e a possibilidade de interações constantes podem assegurar o desenvolvimento da segurança emocional. Promover um cuidado sensível e um apego seguro contribui, portanto, para o desenvolvimento infantil.

Quanto aos temas e conteúdos abordados nos treinamentos para promoção do desenvolvimento infantil com cuidadoras, ressaltam-se informações sobre a relevância da relação cuidador- criança, importância do estabelecimento de vínculo seguro, de cuidados responsivos e prevenção de maus-tratos. Interações sensíveis e responsivas entre o cuidador e a criança representam um elemento fundamental na experiência de crianças institucionalizadas, podendo contribuir para o seu crescimento físico e desenvolvimento^(47)^. Os conteúdos discutidos com os cuidadores estão em conformidade com a estratégia *Nurturing Care*, lançada pela Organização Mundial da Saúde em 2018. Essa estratégia destaca a importância de ações globais direcionadas aos cuidados responsivos às crianças e reconhece o desenvolvimento infantil como um elemento importante para atingir os Objetivos de Desenvolvimento Sustentável (ODS – 2030)^(10)^. O modelo *Nurturing Care,* compreendido em português como “Atenção e Cuidado Integral”, apoia a compreensão das ações necessárias para promoção do desenvolvimento integral em todas as fases da infância, principalmente na primeira infância. De acordo com o modelo de Atenção e Cuidado Integral, as crianças precisam ter cinco componentes inter-relacionados e indivisíveis de cuidado, como boa saúde, nutrição adequada, segurança e proteção, cuidados responsivos, e oportunidades de aprendizado. Entende-se como cuidado responsivo a competência dos cuidadores de perceber, entender e responder aos sinais da criança, de maneira oportuna e apropriada, para atender às suas necessidades^(48)^.

Entre os estudos analisados que realizaram a intervenção para promoção do desenvolvimento infantil de modo presencial, oito deles não relatam se a ação foi realizada individualmente ou em grupo^(13,21,22,23,25,26,30,31)^, enquanto os cinco foram conduzidos em grupo^(20,24,32,33,35)^. Além disso, dois estudos associaram uma supervisão individual posterior ao treinamento em grupo^(25,33)^, demonstrando uma possível preferência dos pesquisadores pela abordagem grupal. Este fato corrobora pesquisas atuais que mostram a prevalência da utilização de abordagens em grupo para discussão da temática com pais e cuidadores, pois estas favorecem a construção de conhecimento entre os participantes por meio da interação, compartilhamento de experiências, e expressão de dificuldades e preocupações^(49,50)^.

Sobre os recursos utilizados nas intervenções, é notável a falta de informações sobre o material da intervenção, uma vez que esses dados estavam disponíveis apenas em dois estudos: um que compartilhou o guia do curso de capacitação e as cartas utilizadas previamente para sensibilização das cuidadoras^(20)^; e outro que inseriu imagens da configuração final do *blog* com informações para as cuidadoras^(27)^. Isso dificulta a análise dos materiais utilizados e não permite que as intervenções sejam replicadas em outros contextos para nova avaliação de sua efetividade.

A dose da intervenção variou entre os estudos, com a falta de descrição do período, duração e frequência em cinco estudos. O menor período relatado foi uma oficina realizada em quatro encontros^(20)^, enquanto a intervenção mais longa ocorreu no período de 2004 a 2008^(34)^. Em relação à duração das oficinas educativas realizadas como intervenção, o tempo foi variado, mas houve uma duração que variou de 90 minutos^(31)^, sendo a sessão mais curta, e oito horas a sessão mais longa^(35)^. Embora haja essa falta de uniformidade na descrição, é importante ressaltar que todos os estudos relataram resultados positivos das intervenções no contexto do desenvolvimento infantil.

A variação na dose da intervenção observada nos estudos destaca a necessidade de uma abordagem mais padronizada e detalhada na descrição dos períodos, duração e frequência das intervenções. Essa uniformidade na descrição é importante porque facilita a comparação entre estudos e permite uma melhor compreensão dos resultados^(45)^. Além disso, uma abordagem padronizada ajuda na replicabilidade das intervenções, permitindo que outros pesquisadores implementem e avaliem as mesmas estratégias em diferentes contextos^(51)^. Isso contribui para o avanço do conhecimento científico sobre intervenções eficazes no desenvolvimento infantil possibilitando a identificação de melhores práticas para promoção do bem-estar das crianças.

De acordo com o manual “Caminhos e aprendizados para iniciativas focadas na primeira infância”, publicado pelo Núcleo Ciência Pela Infância (NCPI), torna-se necessária, para a elaboração de intervenções, uma escuta ativa e empática das demandas, desejos, necessidades e percepções do usuário que irá receber a intervenção, sendo favorável a aproximação por meio de visitas de campo e imersões para se aproximar da realidade e das particularidades do contexto, e aprender com o seu público-alvo^(52)^. O NCPI constitui-se como uma coalizão interinstitucional que envolve centros de pesquisa nacionais e internacionais, como o *Center on the Developing Child* da Universidade Harvard, o Instituto de Ensino e Pesquisa (Insper), a Fundação Maria Cecília Souto Vidigal, pesquisadores da Universidade de São Paulo, entre outros, que atua em estreita colaboração com formuladores de políticas e gestores públicos. Embora não possua *status* normativo, o manual citado tem sido amplamente referenciado em capacitações, programas governamentais e iniciativas do terceiro setor, em razão de sua base empírica e da aplicabilidade em diferentes contextos. Destina-se, em especial, a profissionais das áreas da saúde, educação, assistência social e planejamento urbano que atuam na promoção do desenvolvimento infantil integral.

Complementarmente, a metodologia *IDEAS Impact Framework*, proposta pelo *Frontiers of Innovation* do *Center on the Developing Child* da Universidade de Harvard (2017)^(53)^, estrutura abordagens de pesquisa que visam, em última instância, alcançar intervenções aplicadas em larga escala. Ou seja, essas intervenções devem se expandir para mais pessoas ou beneficiários e se manter por conta própria, conquistando, assim, sua sustentabilidade^(53)^. Ainda de acordo com a metodologia, recomenda-se que a intervenção seja primeiramente aplicada a um pequeno grupo da população-alvo. Com uma amostra reduzida, torna-se mais fácil e rápido realizar ciclos de testes para coletar dados e verificar a praticidade da intervenção. Esses ciclos são fundamentais para obter importantes lições^(53)^. A relevância desse método no campo da ciência da implementação se justifica pela sua capacidade de orientar o desenvolvimento e a avaliação de intervenções na primeira infância com base em evidências. Sua abordagem favorece o ajuste iterativo das ações, assegurando efetividade, escalabilidade e sustentabilidade. Além disso, incorpora práticas sistemáticas de coleta e análise de dados, promovendo o alinhamento das intervenções às necessidades específicas do público-alvo e o aprimoramento contínuo dos mecanismos de mudança^(53)^.

Outro aspecto relevante para a criação de intervenções destinadas à promoção do desenvolvimento infantil é a “cocriação”, ou seja, criar a iniciativa em conjunto com quem vai utilizá-la. Estratégias de escuta, engajamento e interação com os usuários auxiliam na obtenção de uma compreensão mais aprofundada da perspectiva desse público^(53)^.

Os dados coletados para caracterizar as intervenções, recomendados pelo *TIDieR* ^(19)^, revelam uma lacuna na descrição detalhada das intervenções realizadas. Essa ausência de informações compromete a possibilidade de replicação do estudo em outros contextos. Tal situação também foi identificada em revisão de escopo que mapeia intervenções^(54)^ e decorre do fato de que cada intervenção é concebida para atender objetivos específicos, em vez de ser planejada como uma intervenção passível de ser replicada.

Um achado central desta revisão foi a constatação de uma significativa heterogeneidade nas intervenções analisadas, tanto em termos de conteúdo quanto em termos de formato e duração. Essa diversidade metodológica, somada à escassez de informações sobre os materiais utilizados, dose das intervenções e estratégias de implementação, limita a avaliação da efetividade das abordagens e dificulta sua replicação e escalabilidade. A ausência de padronização não apenas fragiliza a base de evidências, mas também representa um obstáculo à formulação de políticas públicas fundamentadas em dados robustos. Portanto, reforça-se a necessidade de futuros estudos adotarem descrições mais sistemáticas e detalhadas das intervenções, conforme recomendações de instrumentos como o *TIDieR*, para que se tornem efetivamente replicáveis, adaptáveis e sustentáveis em variados contextos institucionais.

Os resultados desta revisão demonstram que a capacitação dos cuidadores, mudanças estruturais nos ambientes de cuidado e mudanças no processo de trabalho dos cuidadores apresentam efeitos positivos no desenvolvimento emocional, social e cognitivo das crianças, além de ressaltar a relevância em ampliar os esforços para aprimorar a qualidade dos cuidados nas instituições que acolhem as crianças.

As principais contribuições do estudo decorrem da apresentação de resultados positivos em todas as investigações. Além disso, evidenciou-se a necessidade e relevância de que os relatos de estudos de intervenção contemplem de forma mais abrangente a descrição da intervenção em si, a fim de torná-los replicáveis e escaláveis em outros locais.

Entende-se que o estudo apresenta algumas limitações que devem ser consideradas. Como uma revisão de escopo, os dados obtidos são provenientes de diversos estudos com métodos e modelos diferentes, alguns dos quais podem não ter rigor científico adequado. Além disso, aponta-se a falta de descrição detalhada dos elementos essenciais das intervenções, o que dificulta a compreensão dos resultados e sua comparação, além do baixo número de publicações. Por fim, apesar dos esforços para abranger toda a literatura, alguns dos estudos podem ter sido involuntariamente perdidos, o que pode ter condicionado as conclusões finais e o resumo das evidências apresentadas.

## CONCLUSÃO

As intervenções realizadas com cuidadoras de crianças institucionalizadas para promoção do desenvolvimento infantil foram conduzidas por meio de treinamento e treinamento associado a mudanças estruturais nos serviços, ocorreram de forma predominantemente presencial e em grupo. Os conteúdos trabalhados nas intervenções foram cuidados mais calorosos, sensíveis e responsivos, formação de vínculo seguro entre bebês e seus cuidadores, prevenção de maus-tratos, aquisição de linguagem infantil e a importância do cuidador nesse processo, além de modos de estimulação de pré-escolares com tópicos de aprendizagem. Essas intervenções apresentaram resultados favoráveis e efeitos positivos no desenvolvimento infantil.

Entretanto, observou-se uma grande heterogeneidade entre as intervenções analisadas, com variações de conteúdo, formato e duração, além da escassez de informações sobre os materiais e estratégias utilizados. Essa ausência de padronização dificulta a identificação do que é mais efetivo, limitando a replicação e enfraquece a base de evidências para políticas públicas.

O mapeamento das intervenções realizadas com cuidadores de crianças institucionalizadas atende parcialmente à necessidade de identificar características relevantes para elaboração de futuros programas de intervenções para promoção do desenvolvimento infantil. Nesse sentido, sugere-se a realização de mais estudos que forneçam evidências robustas sobre as características e os resultados das intervenções realizadas com cuidadores de crianças institucionalizadas para promoção do desenvolvimento infantil para orientar a prática. Ressalta-se, ainda, a necessidade de elaboração e implementação gradual de intervenções de alta qualidade sustentáveis e replicáveis com cuidadoras de crianças institucionalizadas, a fim de fortalecer as estruturas institucionais e auxiliar que crianças que vivem nesses espaços tenham maiores possibilidades de alcance do seu potencial máximo de desenvolvimento.

## Data Availability

Todo o conjunto de dados que dá suporte aos resultados deste estudo foi publicado no próprio artigo. Eles foram extraídos de artigos e fontes publicamente disponíveis, conforme detalhado no método e estão disponíveis na tabela de estudos incluída na revisão.

# Translation: Intervenciones para promover el desarrollo infantil de los niños institucionalizados: revisión de alcance

**DOI:** 10.1590/1980-220X-REEUSP-2025-0044es

**Authors:** Vanessa Martins, Vitória da Silva Porto, Katherine Solís-Cordero, Clariana Vitória Ramos de Oliveira, Jéssica Batistela Vicente, Daniela Doulavince Amador, Maria de La Ó Ramallo Veríssimo, Samara Macedo Cordeiro

**Affiliations:** 1 Universidade Estadual de Campinas, Faculdade de Enfermagem, Campinas, SP, Brasil.; 2 Universidad de Costa Rica, Escuela de Enfermería, San José, Costa Rica.; 3 University of Nevada Las Vegas, School of Nursing, Las Vegas, Nevada, United States.; 4 Universidade Federal do Paraná, Coordenadoria de Atenção Integral à Saúde Estudantil, Curitiba, PR, Brasil.; 5 Universidade de São Paulo, Escola de Enfermagem, São Paulo, SP, Brasil.

**Keywords:** Desarrollo Infantil, Niño Institucionalizado, Cuidadores

## Abstract

**Objetivo:**

Describir las características y los resultados de las intervenciones realizadas con cuidadores de niños institucionalizados para promover el desarrollo infantil.

**Resultados:**

Se incluyeron diecisiete estudios que reportaron intervenciones de tipo capacitación, asociadas o no con cambios en el proceso de trabajo y la organización estructural de la institución, así como capacitación mediante la creación de un entorno de aprendizaje virtual. Se observaron efectos positivos en el desarrollo infantil, pero la heterogeneidad de las intervenciones y los resultados impidió identificar sus aspectos más relevantes. El principal contenido abordado fue la atención responsiva.

**Conclusión:**

La diversidad de modelos de capacitación profesional dificulta determinar qué aspectos son más relevantes en la formación de cuidadores, por lo que se requieren estudios más sólidos sobre el tema.

## INTRODUCCIÓN

La primera infancia, de 0 a 6 años^(1)^, es un periodo marcado por vulnerabilidades y oportunidades^(2)^, en el que el niño experimenta cambios físicos, cognitivos y psicosociales, y el cerebro se desarrolla de forma acelerada, por lo que es un momento estratégico para promover su desarrollo. Muchos niños pueden pasar por esta etapa mientras están temporalmente separados de sus familias y/o cuidadores primarios en un centro de acogida institucional hasta que puedan reunirse con ellos o ser derivados a familias sustitutas^(3)^. Según el Consejo Nacional de Justicia, alrededor de 34000 niños viven en situación de acogida institucional en Brasil^(4)^.

El desarrollo infantil (DI) es un proceso activo y único para cada niño, que se expresa a través de la continuidad y los cambios en la adquisición de habilidades motoras, cognitivas, psicosociales y del lenguaje, y los primeros años de vida son la base de este proceso^(5)^. Un cuidado centrado en las necesidades de desarrollo permite al niño alcanzar todo su potencial^(6)^.

Promover el desarrollo de los niños es una prioridad a nivel nacional e internacional^(7)^. En Brasil, el Marco Legal de la Primera Infancia^(1)^ establece como prioridad la creación de programas y políticas públicas que se centren en el desarrollo integral de la primera infancia, además de destacar la importancia de la cualificación de los profesionales, que desempeñan un papel fundamental al proporcionar asistencia directa o apoyar a otros cuidadores, empleando técnicas pedagógicas participativas que estimulen la reflexión y la decisión por comportamientos adecuados a las necesidades individuales de cada niño.

A nivel mundial, la importancia de este tema ha sido respaldada por los Objetivos de Desarrollo Sostenible (ODS)^(8)^, la Estrategia Mundial para la Salud de las Mujeres, los Niños y los Adolescentes (2016–2030)^(9)^ y por *Nurturing Care*, que da prioridad a las intervenciones para promover el desarrollo integral en la primera infancia, incluido el fortalecimiento de las competencias parentales para el cuidado receptivo a las necesidades esenciales del niño^(10)^.

A partir de los conocimientos adquiridos, que demuestran que las interacciones en la primera infancia afectan a los circuitos cerebrales y favorecen el desarrollo emocional e intelectual^(2)^, la promoción del desarrollo infantil y la protección y el cuidado de los niños han dejado de considerarse una responsabilidad exclusiva de sus padres y familiares, pasando a ser objeto de un amplio compromiso político y social. En el contexto de los niños en instituciones de acogida, este compromiso se vuelve aún más esencial, ya que los niños viven experiencias potencialmente adversas, que a menudo implican violencia y posible estrés tóxico.

Las intervenciones destinadas al desarrollo infantil han promovido una ampliación del conocimiento en el campo de la ciencia del desarrollo. Entre las intervenciones, las evidencias indican que el cuidado receptivo, especialmente el que implica métodos de promoción de la interacción entre el cuidador y el niño, es el más eficaz para el desarrollo infantil^(10,11)^. El cuidado receptivo permite satisfacer las necesidades esenciales de los niños, que implican el establecimiento de relaciones saludables y permanentes, la seguridad física, el respeto a las características individuales, experiencias compatibles con la etapa de desarrollo y la convivencia en una comunidad estable y solidaria^(12)^.

Una revisión sistemática^(13)^ identificó que las intervenciones estructurales realizadas en los refugios y las acciones formativas con los cuidadores fueron eficaces para promover el desarrollo infantil. Sin embargo, a pesar de que las evidencias indican la eficacia de estas intervenciones, la literatura aún carece de una descripción detallada de sus características, especialmente en el contexto brasileño. Esta laguna dificulta la replicación, adaptación y ampliación de prácticas basadas en la evidencia, lo que compromete la calidad de la atención prestada.

Teniendo en cuenta que la atención en las instituciones de acogida infantil es prestada por cuidadores no profesionales, cuya formación mínima requerida es la educación secundaria^(14)^, es necesario impartir formación sobre el crecimiento y el desarrollo infantil.

La calidad de la atención y las relaciones entre los niños y sus cuidadores pueden potenciarse mediante intervenciones educativas. Esto justifica conocer y seleccionar la evidencia para elaborar un programa de promoción del desarrollo infantil en el contexto de la institucionalización de niños menores de 6 años. Por lo tanto, el objetivo de este estudio fue mapear las características y los resultados de las intervenciones realizadas con cuidadores de niños institucionalizados para promover el desarrollo infantil.

No se encontraron estudios que permitieran comprender las características de las intervenciones realizadas con cuidadores de niños institucionalizados para promover el desarrollo infantil. La realización de un análisis detallado y exhaustivo de estas intervenciones permite identificar las características que favorecen su implementación, replicación o desarrollo de nuevos estudios basados en los hallazgos identificados.

## MÉTODO

### Tipo de Estudio

Se trata de una revisión de alcance, método que mapea y caracteriza la extensión de las evidencias sobre un campo de investigación, pudiendo evidenciar las lagunas de conocimiento y demostrar la relevancia y necesidad de estudios más profundos sobre determinadas áreas^(15)^. Este estudio se desarrolló y estructuró de acuerdo con las recomendaciones de la JBI^(15)^, tal y como se detalla en los siguientes apartados, y la lista de verificación Preferred Reporting Items for Systematic reviews and Meta-Analyses extension for Scoping Reviews (PRISMA-ScR)^(16)^. El protocolo de la revisión se registró en la plataforma Open Science Framework (https://doi.org/10.17605/OSF.IO/HN8E2).

Las etapas metodológicas del estudio fueron: formulación de la pregunta de investigación, utilizando el mnemotécnico Población, Concepto y Contexto (PCC); definición de los criterios de inclusión y exclusión; definición de los tipos de estudios y fuentes; elaboración de la estrategia de búsqueda; identificación de las bases de datos; búsqueda y selección de estudios; extracción y análisis de datos; y elaboración del informe^(15)^.

### Identificación de la Pregunta de Investigación

La elaboración de la pregunta orientadora se basó en la estructura mnemónica PCC (Población, Concepto y Contexto), siendo Población (P): cuidadores; Concepto (C): intervenciones para la promoción del desarrollo infantil; Contexto (C): institucionalización infantil. Con esta combinación mnemónica, se definió la siguiente pregunta orientadora: ¿cuáles son las características y los resultados de las intervenciones realizadas con cuidadores para promover el desarrollo infantil de niños de 0 a 6 años institucionalizados?

Se consideraron niños institucionalizados aquellos que reciben servicios de larga duración o que residen en un entorno institucional^(17)^, y las intervenciones para promover el desarrollo infantil, acciones que promueven el desarrollo físico, socioemocional, cognitivo y motor, implementadas para niños de 0 a 6 años de edad. Se eligió el rango de edad de 0 a 6 años porque este período corresponde a la primera infancia, una etapa importante para el desarrollo físico, emocional, mental y social del niño, por lo que es estratégico para promover su desarrollo infantil.

### Criterios de Selección

Se incluyeron artículos científicos primarios y revisiones, descriptivas o analíticas, tesis y disertaciones que respondían a la pregunta de la revisión. No hubo limitaciones de tiempo, ubicación geográfica o idioma.

Se excluyeron los estudios que no contemplaban la población, el concepto y el contexto de interés, o que no informaban sobre la aplicación de una intervención, así como editoriales, cartas al editor y artículos de opinión. Además, también se excluyeron los estudios en los que el niño estaba bajo la responsabilidad de una familia de acogida, un programa creado en 2009 cuyo objetivo es enviar a los niños a familias seleccionadas, capacitadas y acompañadas por el gobierno municipal^(18)^, ya que, en estos casos, el niño no se encontraba en un entorno institucional. Los documentos duplicados se consideraron una sola vez.

### Estrategias de Búsqueda

La búsqueda electrónica se realizó entre septiembre y diciembre de 2023 y se actualizó en abril de 2025 con la ayuda de una bibliotecaria utilizando los Descriptores en Ciencias de la Salud (DeCS) y los *Medical Subject Headings* (MeSH), junto con los operadores booleanos *AND* y *OR* en las bases de datos y en la literatura gris (Cuadro 1). También se realizó una búsqueda manual en la lista de referencias de los artículos incluidos.

**Cuadro 1.** Estrategias de búsqueda según bases de datos y literatura gris – São Paulo, SP, Brasil, 2025.

Base de datos y literatura gris	Estrategia de búsqueda
PubMed	(((Child Development[MeSH Terms]) OR (“Child Development”[Title/Abstract] OR “Infant Development”[Title/Abstract])) AND ((Child, Institutionalized[MeSH Terms]) OR (“Child, Institutionalized”[Title/Abstract] OR “Institutionalized Child”[Title/Abstract] OR “Institutionalized Children”[Title/Abstract]))) AND ((Caregivers[MeSH Terms]) OR (Caregivers[Title/Abstract] OR Caregiver[Title/Abstract] OR Carers[Title/Abstract] OR Carer[Title/Abstract] OR “Care Givers”[Title/Abstract] OR “Care Giver”[Title/Abstract] OR “Spouse Caregivers”[Title/Abstract] OR “Spouse Caregiver”[Title/Abstract] OR “Informal Caregivers”[Title/Abstract] OR “Informal Caregiver”[Title/Abstract]))
Biblioteca Virtual en Salud	(“Child Development” OR “Infant Development”) AND (“Child, Institutionalized” OR “Institutionalized Child” OR “Institutionalized Children”) AND (caregivers OR caregiver OR carers OR carer OR “Care Givers” OR “Care Giver” OR “Spouse Caregivers” OR “Spouse Caregiver” OR “Informal Caregivers” OR “Informal Caregiver”)
Scopus	(TITLE-ABS-KEY (“Child Development” OR “Infant Development”)) AND (TITLE-ABS-KEY (“Child, Institutionalized” OR “Institutionalized Child” OR “Institutionalized Children”)) AND (TITLE-ABS-KEY (caregivers OR caregiver OR carers OR carer OR “Care Givers” OR “Care Giver” OR “Spouse Caregivers” OR “Spouse Caregiver” OR “Informal Caregivers” OR “Informal Caregiver”))
Scientific Electronic Library Online	“Child Development” AND “Child, Institutionalized” AND “caregivers”
Cochrane	“Child Development” AND “Child, Institutionalized” AND “caregivers”
Portal de Periódicos da Coordenação de Aperfeiçoamento de Pessoal de Nível Superior	“Child Development” AND “Child, Institutionalized” AND “caregivers”
Web of Science	“Child Development” AND “Child, Institutionalized” AND “caregivers”
PsycINFO	“Child Development” AND “Child, Institutionalized” AND “caregivers”
Google Scholar	“Child Development” AND “Child, Institutionalized” AND “caregivers”
Biblioteca Digital Brasileira de Teses e Dissertações	“Child Development” AND “Child, Institutionalized” AND “caregivers”

### Selección de Estudios

Los resultados obtenidos en todas las plataformas de búsqueda se transfirieron a Rayyan^®^ (*software* utilizado en la selección de estudios en métodos de síntesis de conocimientos) para eliminar los estudios duplicados y realizar la selección de las investigaciones. La selección se llevó a cabo en dos fases.

La primera fase fue realizada por dos revisores independientes capacitados que leyeron y evaluaron los títulos y resúmenes de los registros identificados, con la preselección de los estudios potencialmente elegibles. En caso de desacuerdo, se llamó a un tercer revisor para decidir sobre la inclusión o no de los artículos. El tercer revisor tenía experiencia en el tema del desarrollo infantil y en la realización de revisiones sistemáticas de la literatura. En la segunda fase, llevada a cabo por una de las revisoras, se evaluó el texto completo de los estudios preseleccionados para confirmar su elegibilidad.

### Extracción y Síntesis de los Datos

Una vez definida la muestra de estudios incluidos, se extrajeron los datos en un formulario específico para la identificación del estudio (título, autor, país, año de publicación, tipo de estudio y objetivo). Los datos extraídos para la caracterización de las intervenciones fueron población, tipo de intervención, contexto de realización de las intervenciones y dominio del desarrollo, además de los recomendados por la Plantilla para la Descripción y Replicación de Intervenciones (*Template for Intervention Description and Replication* – TIDieR)^(19)^, como referencia teórica utilizada, profesional responsable de la intervención, contenido de la intervención, materiales y estrategias utilizados, modo de entrega, dosis (número de veces, duración y período) y resultados de la intervención. El *TIDieR* tiene como objetivo mejorar la descripción detallada de las intervenciones para permitir su replicabilidad.

Esta información se introdujo en una hoja de cálculo de *Microsoft Office World* ^®^ y permitió la síntesis e interpretación de los estudios.

### Aspectos Éticos

Al tratarse de una investigación de revisión, no fue necesaria la evaluación del Comité de Ética e Investigación para su desarrollo.

## RESULTADOS

Inicialmente se identificaron un total de 417 estudios, de los cuales se excluyeron 274 duplicados mediante la plataforma Rayyan^®^, lo que dio como resultado 143 estudios únicos. De estos, 115 fueron excluidos tras la evaluación de los títulos y resúmenes, y 28 fueron sometidos a una lectura completa. En la fase de lectura completa, se excluyeron 17 artículos por no abordar las características y los resultados de las intervenciones realizadas con cuidadores para promover el desarrollo infantil de niños de 0 a 6 años institucionalizados, y se incluyeron 11 estudios en la investigación. Mediante la búsqueda manual en la lista de referencias, se identificaron seis nuevos estudios, lo que dio como resultado una muestra final de 17 estudios (Figura 1).

**Figura 1:** Flujograma del proceso de selección de los artículos para la revisión de alcance adaptado de *PRISMA-ScR 2020 flow diagram for new systematic reviews which included searches of databases, registers and other sources*. São Paulo, SP, Brasil, 2025.

En cuanto al tipo de estudio, tal y como se describe en los artículos, se identificaron estudios cualitativos^(20)^, revisiones bibliográficas^(13,21,22)^, estudios de intervención cuasi-experimental^(23,24)^, estudios de cohorte^(25,26)^, estudios metodológicos^(27)^, capítulos de libros^(28)^, estudios de intervención no aleatorios^(29,30)^, estudios longitudinales prospectivos^(31)^, intervenciones piloto^(32,33)^, estudios de caso^(34)^ y estudios de viabilidad^(35)^. Las investigaciones se publicaron entre 2002 y 2021, y se llevaron a cabo en Brasil^(20,27)^, Estados Unidos^(13,21,22,28,32)^, Rusia^(23,25,26,29)^, Chile^(24)^, India^(31)^, Turquía^(30)^, Nepal^(34)^, Tanzania^(35)^ y los países de América Central^(33)^.

En el Cuadro 2 se presentan los objetivos y resultados de las intervenciones.

**Cuadro 2.** Identificación de los estudios según autor, país, año de publicación, objetivo del estudio y resultados – São Paulo, SP, Brasil, 2025.

Título	Autor País Año	Objetivo	Resultado
Cuidadoras de crianças institucionalizadas: intervenção e cuidados	Jacqueline Müllich Fensterseifer Brasil 2021	Presentar los conocimientos adquiridos sobre cómo se atiende a los bebés en situación de acogida.	Las intervenciones contribuyeron a promover vínculos afectivos de mejor calidad entre los cuidadores y los niños. Se observaron mejoras en la salud mental y el bienestar de los cuidadores y los niños.
Early Caregiver–Child Interaction and Children’s Development: Lessons from the St. Petersburg-USA Orphanage Intervention Research Project	Robert B McCall *et al.* EEEUU 2018	Revisar los estudios que tratan sobre el desarrollo de los niños que residen en instituciones en la Federación Rusa o que han sido acogidos por familias en los Estados Unidos y Rusia.	La mejora de las interacciones entre los cuidadores y los niños en las instituciones tiene un impacto positivo en el desarrollo físico y conductual de los niños. Las cuidadoras mejoraron su respuesta de cuidado ante el llanto de los niños.
Caregiver-child Interaction, Caregiver Transitions, And Group Size As Mediators Between Intervention Condition And Attachment And Physical Growth Outcomes In Institutionalized Children.	Hilary A Warner *et al.* Rusia 2017	Describir el análisis secundario de los datos del estudio de intervención ruso para determinar si la calidad de la interacción entre el cuidador y el niño, las transiciones entre cuidadores y el tamaño del grupo influyen en el efecto de la intervención en el apego y el crecimiento físico de los niños.	Se observó una mejora en la calidad de las interacciones entre las cuidadoras y los niños. Los niños que vivían en las instituciones sometidas a la intervención obtuvieron una estatura, un peso y un perímetro craneal significativamente mayores que los niños que no fueron sometidos a la intervención.
Effects of an intervention to promote socioemotional development in terms of attachment security: a study in early institutionalization in Chile	Felipe Lecannelier *et al.* Chile 2014	Evaluar el estado psicoafectivo de los bebés en instituciones en las áreas de apego, desarrollo psicomotor, temperamento y desarrollo social, además de analizar la eficacia de un programa de promoción del desarrollo socioemocional implementado en los centros residenciales, considerando la seguridad del apego como variable organizadora.	Se observó una mejora en la relación emocional entre el niño y el cuidador, en la capacidad de atención y en la seguridad del apego, con un aumento en el porcentaje de bebés con apego seguro.
Fostering Child Development by Improving Care Quality: A Systematic Review of the Effectiveness of Structural Interventions and Caregiver Trainings in Institutional Care	Katharin Hermenau *et al.* EEUU 2016	Revisar sistemáticamente la evidencia actual sobre la eficacia de las intervenciones estructurales y la formación de los cuidadores en los centros de acogida, con el fin de promover el desarrollo de los niños mediante la mejora de la calidad de la atención y la prevención del maltrato.	La calidad del cuidado infantil, especialmente durante los primeros años de vida, tiene un impacto crucial en el desarrollo y el funcionamiento mental saludable en etapas posteriores. Se han observado efectos beneficiosos en el desarrollo emocional, social y cognitivo de los niños en entornos institucionales.
Caregiver sensitivity and consistency and children’s prior family experience as contexts for early development within institutions	Brandi N Hawk *et al.* Rusia 2018	Examinar la relación entre la sensibilidad del cuidador y la coherencia con el desarrollo socioemocional y cognitivo de los niños pequeños institucionalizados.	Mejorar la sensibilidad y la coherencia de los cuidadores puede tener un impacto positivo en el desarrollo cognitivo y socioemocional de los niños institucionalizados, especialmente durante el primer año.
Maintaining a Social-Emotional Intervention and its Benefits for Institutionalized Children	Robert B McCall *et al.* Rusia 2013	Informar sobre el mantenimiento de una intervención socioemocional llevada a cabo en orfanatos rusos utilizando al equipo de cuidadores habituales.	La intervención de capacitación y cambio estructural mostró mejoras en el desarrollo durante y después de la intervención; sin embargo, la rotación de personal puede perjudicar su continuidad y afectar la consistencia de los cuidadores, lo que repercute en los resultados del desarrollo de los niños.
Estimulação de linguagem para cuidadores de crianças institucionalizadas: elaboração de um blog	Maria Gabriela Cavalheiro; Simone Aparecida Lopes-Herrera Brasil 2019	Crear un entorno virtual de aprendizaje en el ámbito de la estimulación del lenguaje destinado a la formación de cuidadores de niños institucionalizados en refugios, en un contexto de educación a distancia.	Los resultados destacaron el potencial de las acciones de educación a distancia en la capacitación de profesionales relacionados con la población en riesgo de sufrir alteraciones del lenguaje.
Earlier is better: a meta-analysis of 70 years of intervention improving cognitive development in institutionalized children	Marian J. Bakermans-Kranenburg, Marinus H. Van IJzendoorn, Femmie Juffer EEUU 2008	Revisar los estudios de intervención orientados a mejorar el desarrollo de los niños institucionalizados.	Las intervenciones realizadas en instituciones muestran mejoras significativas en el desarrollo cognitivo, del lenguaje y socioemocional de los niños. Además, algunas intervenciones también han beneficiado su salud física, su crecimiento y su desarrollo motor.
Young Children in Institutional Care: Characteristics of Institutions, Children’s Development, and Interventions in Institutions	Megan M Julian *et al.* EEUU 2019	Describir los entornos sociales de instituciones de todo el mundo y las intervenciones realizadas en esos lugares.	Mejorar la calidad de la atención en las instituciones puede tener un impacto significativo en el desarrollo de los niños que residen en ellas, especialmente cuando los cuidadores interactúan regularmente con los mismos niños, aumentando así su sensibilidad y capacidad de respuesta durante las actividades diarias.
The role of transitions to new age groups in the development of institutionalized children	Robert B McCall *et al.* Rusia 2012	Describir los efectos de la transición a nuevos grupos de edad y cuidadores en el desarrollo general de los niños institucionalizados.	La rotación de habitaciones, cuidadores y grupos puede ser perjudicial para los niños institucionalizados, pero se mitiga mediante una interacción sensible entre el cuidador y el niño. La capacitación para mejorar esta interacción y evitar transiciones ha dado lugar a un desarrollo más positivo y menos estresante.
‘Not by bread alone’: impact of a structured 90-minute play session on development of children in an orphanage	V. Taneja *et al.* India 2002	Desarrollar un programa de intervención de juego estructurado.	Los niños mostraron una mayor capacidad de respuesta, un mejor control de la cabeza y el cuerpo, y se mostraron más activos. Las cuidadoras se mostraron reacias al inicio de la intervención debido al aumento de la carga de trabajo, pero al final se mostraron entusiasmadas.
A socioemotional intervention in a Latin American orphanage	Robert B McCall *et al.* EEUU 2012	Evaluar cómo una intervención socioemocional que enfatizó la capacitación y la asistencia técnica podría afectar el desarrollo de los niños institucionalizados.	Tras la intervención, se observó una mejora significativa en las interacciones entre los cuidadores y los niños, con un enfoque más cálido, atento, sensible y participativo, especialmente hacia los niños más pequeños. Las mejoras fueron más pronunciadas durante actividades como la alimentación, el baño/cambio de ropa y los juegos libres.
Environmental Enrichment and Caregiver Training to Support the Development of Birth to 6-Year-Olds in Turkish Orphanages	Sibel Kazak Berument Turquía 2013	Desarrollar un programa de intervención que integre el enriquecimiento ambiental y la capacitación de los cuidadores, con el objetivo de mejorar el lenguaje y el desarrollo cognitivo de los bebés y niños pequeños que residen en entornos institucionales en Turquía.	El programa de intervención fue eficaz para mejorar la calidad de la atención en los orfanatos, y también tuvo un efecto en el desarrollo lingüístico y cognitivo de los bebés y niños en edad preescolar que participaron en este estudio.
From maid to mother: transforming facilities, staff training, and caregiver dignity in an institutional facility for young children in Nepal	Amy Conley Wright *et al.* Nepal 2014	Informar sobre la mejora en salud, la seguridad y el desarrollo de los niños de 0 a 6 años en un orfanato de Nepal.	Se observó una disminución de las enfermedades infecciosas y un aumento de las interacciones sociales entre los niños y los cuidadores. Además, se observó un avance en el desarrollo cognitivo y emocional de los niños tras la implementación de las intervenciones, junto con una mejora en la autoestima y el sentido de “dignidad” entre los cuidadores.
Improving care quality and preventing maltreatment in institutional care – a feasibility study with caregivers	Katharin Hermenau *et al.* Tanzania 2015	Probar la viabilidad de una intervención para promover el desarrollo infantil y evaluar los cambios en los índices de maltrato infantil.	La formación es viable incluso en circunstancias difíciles. El contenido abordado es aplicable al proceso de trabajo del cuidador. Los niños también informaron de una mejora en los cuidados.
The Effects of a Social–Emotional Intervention on Caregivers and Children With Disabilities in Two Central American Institutions	Christina J Groark *et al.* América Central 2013	Informar sobre una intervención piloto para mejorar dos instituciones centroamericanas que atendían a niños con discapacidades graves y múltiples utilizando un enfoque de formación de formadores para animar a los cuidadores habituales del personal a interactuar con los niños de forma sensible, receptiva y respetuosa.	Las intervenciones que priorizan el cuidado sensible y receptivo, junto con algunas técnicas básicas especializadas de posicionamiento y manejo, pueden aplicarse con éxito para mejorar el desarrollo físico y conductual/mental de los niños institucionalizados con discapacidades graves y múltiples.

Sigla: EEUU – Estados Unidos de América.

Todos los estudios se realizaron con cuidadoras de niños institucionalizados, en su mayoría en la propia institución de acogida, y en uno de los estudios, la intervención de tipo formación se llevó a cabo con cuidadoras de escuelas primarias de la región^(35)^. La intervención se llevó a cabo de forma virtual en un estudio^(27)^, en trece de forma presencial^(13,20,21,22,23,24,25,26,29,30,31,33,35)^, y en tres estudios no se describe esta información^(28,29,34)^. Entre las intervenciones presenciales, cinco se llevaron a cabo en grupos^(20,24,29,33,35)^ y ocho^(13,21,22,23,25,26,28,30)^ no especifican si la intervención se realizó en grupo o de forma individual. Dos estudios mencionan que, además de realizar una capacitación en grupo, se llevó a cabo una supervisión individual de los cuidadores después de la capacitación^(25,33)^.

En cuanto al uso de referencias teóricas para la construcción de la intervención: un estudio^(20)^ utilizó documentos de las áreas de salud, psicología social, psicoanálisis y asistencia social, el Estatuto del Niño y del Adolescente y las Orientaciones Técnicas para los Servicios de Acogida de Niños y Adolescentes de 2009; tres^(24,25,29)^ utilizaron la Teoría del Apego de John Bowlby; uno^(31)^ utilizó la Teoría Social del Aprendizaje; uno^(32)^ utilizó un programa ya conocido para el cuidado infantil en asociación con los preceptos del Instituto Pikler de Budapest; uno^(35)^ siguió las directrices de la Academia Americana de Pediatría (1999) y conceptos de formación preestablecidos; y diez estudios^(13,22,23,25,26,27,28,30,33,34)^ no mencionaron ningún marco teórico en la elaboración de la intervención.

Los tipos de intervención descritos fueron: capacitación de los cuidadores en acciones que promueven el desarrollo infantil durante el cuidado^(13,20,24,33)^; capacitación asociada a cambios en el proceso de trabajo y en la organización estructural de la institución^(21,23,25,26,28,29,30,32,34)^; y capacitación mediante la creación de un entorno virtual de aprendizaje^(27)^. Uno de los estudios utilizó una técnica de modelado, o imitación, en la que se animaba a los cuidadores a repetir las acciones de los agentes de la intervención^(31)^, y otro estudio, además de la capacitación y los cambios estructurales, ofreció asistencia técnica de seguimiento a los cuidadores^(32)^.

Las intervenciones se centraron en los ámbitos del desarrollo socioemocional^(13,20,21,22,23,24,25,26,28,29,32,34,35),^ motor^(21,23,26,31,32)^, cognitivo^(13,21,22,23,26,30,31,32,33,34)^, el lenguaje^(20,22,27,30,31,32)^ y físico^(33,34)^.

Los materiales utilizados para la intervención variaron entre cartas y objetos cotidianos de un niño^(20)^, juguetes y materiales de apoyo como cambiadores y camas^(33)^, un manual de sensibilidad en el apego^(24)^, juguetes, libros y canciones pertinentes a cada grupo de edad^(13,26,30,31,32)^, registro electrónico en la *web* con contenidos teórico-prácticos^(27)^, libros y DVD^(29)^. Siete estudios no describieron los materiales utilizados en la intervención^(21,22,23,25,28,34,35)^.

Los profesionales responsables de la intervención fueron psicólogos^(20,31)^, psicólogos con el apoyo de enfermeros^(35)^ y un equipo multidisciplinario, con la presencia de enfermeros^(21,25)^ y logopedas^(27)^. En uno de los estudios, los responsables fueron profesionales de dos empresas dedicadas al desarrollo infantil, una de ellas sin fines de lucro, además de contar con el apoyo de una asistente social y una psicóloga^(29)^. Siete estudios no especifican el profesional responsable de la intervención^(13,22,23,24,26,28,29)^. Un estudio solo informa que el profesional recibió una formación previa de posgrado para implementar la intervención^(33)^.

En cuanto a la dosis, los estudios variaron en la distribución de sus intervenciones. En cuatro estudios no se describe el período de realización, la duración y la frecuencia^(23,26,28,29)^. Una investigación realizó la intervención en 90 horas de observación y talleres realizados en cuatro encuentros^(20)^. Otras intervenciones variaron entre 12 y 14 encuentros^(21)^; la capacitación se realizó en cuatro horas^(24)^; hubo capacitaciones con nueve módulos y 11 sesiones de tres horas para cada módulo^(25)^; hubo un año de capacitación semanal con una duración de tres horas por sesión^(34)^; hubo capacitación durante dos semanas, ocho horas al día y seis días a la semana^(35)^; y hubo nueve sesiones mensuales de ocho horas, complementadas con mini-sesiones mensuales de una a tres horas^(33)^. Una intervención se dividió en dos fases, la primera de las cuales tuvo lugar en un mes y la segunda en dos meses, y cada sesión contenida en estas fases duraba 90 minutos^(31)^. Otra intervención se llevó a cabo semanalmente durante un año^(32)^. Una intervención se dividió en una capacitación que se llevó a cabo durante 17 semanas con reuniones quincenales de 90 minutos de duración cada una y una fase de supervisión de actividades, en la que se planificaron ocho reuniones, pero solo se llevaron a cabo dos^(30)^.

Los principales contenidos trabajados en las intervenciones fueron la aplicación de cuidados amorosos, sensibles y receptivos^(21,23,25,26,28,29,30,32,33)^, la formación de un vínculo seguro entre los bebés y sus cuidadores^(24,29,30)^, la prevención del maltrato^(35)^, la adquisición del lenguaje infantil y la importancia del cuidador en este proceso^(27)^. Además, también se abordó la estimulación de los niños en edad preescolar con temas de aprendizaje como los colores, los números y las estaciones del año^(30)^. Otro enfoque de la intervención fue la mejora del trabajo diario y la mejora de las habilidades en el momento del cuidado^(34)^.

Los cambios en el proceso de trabajo incluyeron la reducción del número de cuidadoras por grupo de niños, la reducción del número de niños por grupo para el cuidado, el aumento de la consistencia del cuidado de una cuidadora principal y la creación de la «hora de la familia», un tiempo de calidad sin distracciones e interferencias externas entre las cuidadoras y los niños^(21,23,26,28,29)^. Uno de los estudios proporcionó una mejora en el menú, la higiene de los materiales y el saneamiento, y también instituyó visitas regulares de médicos y enfermeras para la atención de la salud^(34)^.

## DISCUSIÓN

Esta revisión de alcance evidenció que las intervenciones dirigidas a la capacitación de los cuidadores de niños institucionalizados tienen el potencial de promover mejoras significativas en los ámbitos socioemocional, motor, cognitivo, lingüístico y físico de los niños involucrados en el contexto institucional.

El uso de diferentes tipos de intervenciones puede conducir a resultados de implementación más o menos exitosos. Los estudios muestran que algunos métodos de capacitación, como los talleres presenciales sin seguimiento, no aumentan la adquisición de habilidades por parte del cuidador^(36,37)^, mientras que los métodos de formación más intensivos pueden conducir a ganancias significativas en las competencias de los proveedores de servicios.

Un estudio que evaluó la capacitación de terapeutas en habilidades psicosociales encontró evidencia de que los talleres con seguimiento adicional y las capacitaciones «multicomponentes» que incluían observación, feedback y coaching aumentaron la adquisición de habilidades^(36)^. Sin embargo, aún no está claro si los métodos de formación más rigurosos que pueden mejorar los resultados de los cuidadores, también mejoran os resultados de la población atendida^(38)^.

Un estudio realizado en Australia con terapeutas ocupacionales identificó la preferencia por un apoyo individual después de la formación para implementar el programa enseñado en la práctica, lo que confirma los datos de la literatura que indican que el suministro de nueva información por sí solo tiene un impacto limitado en el cambio de comportamiento^(39)^.

Otro estudio, realizado con asistentes escolares, demostró que la combinación de un enfoque contextual y el coaching individual después de un taller es más eficaz que la realización de un solo taller para ME el desempeño de estos profesionales^(40)^. El coaching se define como una interacción de aprendizaje en la que un especialista observa el desempeño de otra persona y le ofrece feedback objetivo y formativo con el fin de promover la autorreflexión sobre el desempeño para alcanzar los objetivos previamente establecidos^(41)^.

Los estudios demuestran que el seguimiento de las actividades y los comportamientos del cuidador, además del fomento de la adopción de prácticas cualificadas, es crucial para la sostenibilidad de la intervención y los cambios en la promoción del desarrollo infantil^(32)^.

La sostenibilidad se refiere a la continuidad de los efectos positivos de las intervenciones y al mantenimiento de sus efectos a largo plazo^(42)^. Según la literatura, otros factores que pueden influir en la sostenibilidad se refieren a la adaptación, caracterizada por la flexibilidad para realizar transformaciones a lo largo del tiempo según el contexto, y al aprendizaje organizacional, que implica la formación continua y la baja rotación del personal. Además, el apoyo organizacional y las asociaciones son esenciales para crear un entorno interno favorable. La integración con la misión institucional y la presencia de «defensores» que puedan sostener las intervenciones en períodos vulnerables también son cruciales. Además, la financiación debe ser continua y adecuada, procedente de diversas fuentes, y el mantenimiento de los beneficios depende de la percepción de los participantes de que la intervención está logrando los resultados inicialmente deseados^(42)^. En una revisión sistemática de la literatura, se observa que las intervenciones que incluyen componentes de capacitación (tanto profesional como no profesional) tienden a ser más sostenibles que aquellas que no incorporan tales actividades. Las personas capacitadas tienen la capacidad de seguir proporcionando beneficios al capacitar a miembros de la comunidad, estableciendo una red de apoyo al programa^(43)^.

Entre las intervenciones analizadas, destaca la realización de capacitaciones con los cuidadores asociadas a cambios estructurales, y los resultados apuntan a una mejora en la calidad de las interacciones entre cuidadores y niños en estas instituciones, mejores tasas de desarrollo infantil y sensibilización sobre la importancia de los cuidados receptivos. Estos hallazgos destacan los efectos positivos de las intervenciones que combinan la capacitación con cambios estructurales, lo que proporciona una base sólida para futuras iniciativas destinadas a promover el desarrollo infantil.

Además, estos estudios subrayan la necesidad continua de invertir en programas de capacitación para cuidadores, con el fin de mejorar la calidad de los cuidados prestados a los niños^(44)^. Una investigación realizada en Tanzania con niños menores de 18 meses de edad constató que las intervenciones que fomentan la capacitación y la participación activa de los cuidadores y los niños, con el objetivo de promover el desarrollo infantil, tuvieron un impacto significativo en el desarrollo cognitivo y lingüístico^(44)^. La evidencia de que las intervenciones que combinan la capacitación con cambios estructurales dan lugar a mejoras significativas en las interacciones entre el cuidador y el niño y en el desarrollo infantil destaca la importancia de los enfoques integrados y las intervenciones con múltiples componentes en la promoción del bienestar infantil^(44,45)^. Sin embargo, es fundamental reconocer que el éxito de estas intervenciones depende no solo de una implementación eficaz, sino también del apoyo continuo y la participación de las comunidades e instituciones involucradas. Por lo tanto, las investigaciones y políticas futuras deben centrarse en garantizar la sostenibilidad de estos programas, así como en adaptarlos a las necesidades específicas de los diferentes contextos culturales y sociales.

Al elaborar las intervenciones con los cuidadores, entre los estudios que presentaron un marco teórico, la teoría más utilizada fue la teoría del apego de John Bowlby. Para Bowlby, el apego se considera un mecanismo innato en los seres humanos, ya que el individuo está biológicamente programado para reconocer la presencia de una figura disponible que ofrezca una relación de intercambio y proporcione una sensación de seguridad^(46)^. Por lo general, la figura de apego para el niño es la madre biológica; sin embargo, en el caso de los niños institucionalizados, se rompe este paradigma, ya que se produce una ruptura momentánea o continua de los lazos familiares y, por lo tanto, las cuidadoras pasan a desempeñar ese papel^(46)^.

Las herramientas necesarias para que los niños alcancen su potencial intelectual, social, emocional y físico se proporcionan mediante la atención a las necesidades esenciales^(12)^. La primera necesidad que debe satisfacerse es la de relaciones continuas y de apoyo, en las que la presencia de una persona principal en el cuidado del niño, es decir, un adulto de referencia, y la posibilidad de interacciones constantes pueden garantizar el desarrollo de la seguridad emocional.

Por lo tanto, promover un cuidado sensible y un apego seguro contribuye al desarrollo infantil. En cuanto a los temas y contenidos abordados en las capacitaciones para la promoción del desarrollo infantil con las cuidadoras, se destaca la información sobre la relevancia de la relación cuidador-niño, la importancia de establecer un vínculo seguro, el cuidado receptivo y la prevención del maltrato.

Las interacciones sensibles y receptivas entre el cuidador y el niño representan un elemento fundamental en la experiencia de los niños institucionalizados, ya que pueden contribuir a su crecimiento físico y desarrollo^(47)^. Los contenidos discutidos con los cuidadores están en consonancia con la estrategia *Nurturing Care*, lanzada por la Organización Mundial de la Salud en 2018. Esta estrategia destaca la importancia de las acciones globales dirigidas al cuidado receptivo de los niños y reconoce el desarrollo infantil como un elemento importante para alcanzar los Objetivos de Desarrollo Sostenible (ODS – 2030)^(10)^. El modelo *Nurturing Care,* entendido en español como «Atención y Cuidado Integral», apoya la comprensión de las acciones necesarias para promover el desarrollo integral en todas las etapas de la infancia, especialmente en la primera infancia. Según el modelo de Atención y Cuidado Integral, los niños necesitan cinco componentes interrelacionados e indivisibles de cuidado, como buena salud, nutrición adecuada, seguridad y protección, cuidados receptivos y oportunidades de aprendizaje. Se entiende por cuidado receptivo la capacidad de los cuidadores para percibir, comprender y responder a las señales del niño, de manera oportuna y adecuada, para satisfacer sus necesidades^(48)^.

Entre los estudios analizados que realizaron la intervención para promover el desarrollo infantil de manera presencial, ocho de ellos no informan si la acción se llevó a cabo de manera individual o en grupo^(13,21,22,23,25,26,30,31)^, mientras que los cinco se realizaron en grupo^(20,24,32,33,35)^. Además, dos estudios asociaron una supervisión individual posterior a la capacitación en grupo^(25,33)^, lo que demuestra una posible preferencia de los investigadores por el enfoque grupal. Este hecho corrobora investigaciones actuales que muestran la prevalencia del uso de enfoques grupales para discutir el tema con padres y cuidadores, ya que estos favorecen la construcción de conocimiento entre los participantes a través de la interacción, el intercambio de experiencias y la expresión de dificultades y preocupaciones^(49,50)^.

En cuanto a los recursos utilizados en las intervenciones, es notable la falta de información sobre el material de la intervención, ya que estos datos solo estaban disponibles en dos estudios: uno que compartió la guía del curso de capacitación y las cartas utilizadas previamente para sensibilizar a las cuidadoras^(20)^; y otro que insertó imágenes de la configuración final del *blog* con información para las cuidadoras^(27)^. Esto dificulta el análisis de los materiales utilizados y no permite que las intervenciones se repliquen en otros contextos para una nueva evaluación de su eficacia.

La dosis de la intervención varió entre los estudios, con la falta de descripción del período, la duración y la frecuencia en cinco estudios. El período más corto reportado fue un taller realizado en cuatro sesiones^(20)^, mientras que la intervención más larga se llevó a cabo en el período de 2004 a 2008^(34)^. En cuanto a la duración de los talleres educativos realizados como intervención, el tiempo fue variable, pero hubo una duración que varió de 90 minutos^(31)^, siendo la sesión más corta, a ocho horas, la sesión más larga^(35)^. Aunque existe esta falta de uniformidad en la descripción, es importante destacar que todos los estudios informaron resultados positivos de las intervenciones en el contexto del desarrollo infantil.

La variación en la dosis de la intervención observada en los estudios destaca la necesidad de un enfoque más estandarizado y detallado en la descripción de los períodos, la duración y la frecuencia de las intervenciones. Esta uniformidad en la descripción es importante porque facilita la comparación entre estudios y permite una mejor comprensión de los resultados^(45)^. Además, un enfoque estandarizado ayuda a la replicabilidad de las intervenciones, permitiendo que otros investigadores implementen y evalúen las mismas estrategias en diferentes contextos^(51)^. Esto contribuye al avance del conocimiento científico sobre intervenciones eficaces en el desarrollo infantil, permitiendo la identificación de las mejores prácticas para promover el bienestar de los niños.

Según el manual “Caminhos e aprendizados para iniciativas focadas na primeira infância” (Caminos y aprendizajes para iniciativas centradas en la primera infancia), publicado por el Núcleo Ciência Pela Infância (NCPI), para la elaboración de intervenciones es necesario escuchar de manera activa y empática las demandas, deseos, necesidades y percepciones del usuario que recibirá la intervención, siendo favorable el acercamiento mediante visitas de campo e inmersiones para acercarse a la realidad y las particularidades del contexto, y aprender con su público objetivo^(52)^. El NCPI es una coalición interinstitucional que involucra a centros de investigación nacionales e internacionales, como el Centro para el Desarrollo Infantil de la Universidad de Harvard, el Instituto de Enseñanza e Investigación (Insper), la Fundación Maria Cecília Souto Vidigal, investigadores de la Universidad de São Paulo, entre otros, que trabaja en estrecha colaboración con los formuladores de políticas y los gestores públicos. Aunque no tiene *estatus* normativo, el manual citado ha sido ampliamente referenciado en capacitaciones, programas gubernamentales e iniciativas del tercer sector, debido a su base empírica y su aplicabilidad en diferentes contextos. Está destinado, en especial, a profesionales de las áreas de salud, educación, asistencia social y planificación urbana que trabajan en la promoción del desarrollo infantil integral.

De manera complementaria, la metodología *IDEAS Impact Framework*, propuesta por *Frontiers of Innovation* del *Center on the Developing Child* de la Universidad de Harvard (2017)^(53)^, estructura enfoques de investigación que, en última instancia, tienen como objetivo lograr intervenciones aplicadas a gran escala. Es decir, estas intervenciones deben expandirse a más personas o beneficiarios y mantenerse por sí mismas, logrando así su sostenibilidad^(53)^. Según la metodología, se recomienda que la intervención se aplique primero a un pequeño grupo de la población objetivo. Con una muestra reducida, resulta más fácil y rápido realizar ciclos de pruebas para la recolección de datos y verificar la viabilidad de la intervención. Estos ciclos son fundamentales para obtener lecciones importantes^(53)^. La relevancia de este método en el campo de la ciencia de la implementación se justifica por su capacidad para orientar el desarrollo y la evaluación de intervenciones en la primera infancia basadas en la evidencia. Su enfoque favorece el ajuste iterativo de las acciones, lo que garantiza la eficacia, la escalabilidad y la sostenibilidad. Además, incorpora prácticas sistemáticas de recolección y análisis de datos, lo que promueve la alineación de las intervenciones con las necesidades específicas del público objetivo y la mejora continua de los mecanismos de cambio^(53)^.

Otro aspecto relevante para la creación de intervenciones destinadas a promover el desarrollo infantil es la “co-creación”, es decir, crear la iniciativa junto con quienes la van a utilizar. Las estrategias de escucha, compromiso e interacción con los usuarios ayudan a obtener una comprensión más profunda de la perspectiva de este público^(53)^.

Los datos recolectados para caracterizar las intervenciones, recomendados por el *TIDieR* ^(19)^, revelan una laguna en la descripción detallada de las intervenciones realizadas. Esta falta de información compromete la posibilidad de replicar el estudio en otros contextos. Esta situación también se identificó en una revisión del alcance que mapea las intervenciones^(54)^ y se debe al hecho de que cada intervención está diseñada para cumplir objetivos específicos, en lugar de estar planificada como una intervención susceptible de ser replicada.

Un hallazgo central de esta revisión fue la constatación de una heterogeneidad significativa en las intervenciones analizadas, tanto en términos de contenido como de formato y duración. Esta diversidad metodológica, sumada a la escasez de información sobre los materiales utilizados, la dosis de las intervenciones y las estrategias de implementación, limita la evaluación de la eficacia de los enfoques y dificulta su replicación y escalabilidad. La falta de estandarización no solo debilita la base empírica, sino que también representa un obstáculo para la formulación de políticas públicas basadas en datos sólidos. Por lo tanto, se refuerza la necesidad de que los estudios futuros adopten descripciones más sistemáticas y detalladas de las intervenciones, de acuerdo con las recomendaciones de instrumentos como el *TIDieR*, para que sean efectivamente replicables, adaptables y sostenibles en diversos contextos institucionales.

Los resultados de esta revisión demuestran que la capacitación de los cuidadores, los cambios estructurales en los entornos de cuidado y los cambios en el proceso de trabajo de los cuidadores tienen efectos positivos en el desarrollo emocional, social y cognitivo de los niños, además de resaltar la importancia de ampliar los esfuerzos para mejorar la calidad de la atención en las instituciones que acogen a los niños.

Las principales contribuciones del estudio se derivan de la presentación de resultados positivos en todas las investigaciones. Además, se evidenció la necesidad y la importancia de que los informes de los estudios de intervención contemplen de manera más amplia la descripción de la intervención en sí, con el fin de que sean replicables y escalables en otros lugares.

Se entiende que el estudio presenta algunas limitaciones que deben tenerse en cuenta. Al tratarse de una revisión de alcance, los datos obtenidos proceden de diversos estudios con métodos y modelos diferentes, algunos de los cuales pueden no tener la rigurosidad científica adecuada. Además, se señala la falta de una descripción detallada de los elementos esenciales de las intervenciones, lo que dificulta la comprensión de los resultados y su comparación, además del escaso número de publicaciones. Por último, a pesar de los esfuerzos por abarcar toda la bibliografía, es posible que algunos de los estudios se hayan perdido involuntariamente, lo que puede haber condicionado las conclusiones finales y el resumen de las evidencias presentadas.

## CONCLUSIÓN

Las intervenciones realizadas con cuidadoras de niños institucionalizados para promover el desarrollo infantil se llevaron a cabo mediante capacitación y capacitación asociada a cambios estructurales en los servicios, y se realizaron predominantemente de forma presencial y en grupo. Los contenidos trabajados en las intervenciones fueron cuidados más cálidos, sensibles y receptivos, la formación de un vínculo seguro entre los bebés y sus cuidadores, la prevención del maltrato, la adquisición del lenguaje infantil y la importancia del cuidador en este proceso, además de formas de estimulación de los niños en edad preescolar con temas de aprendizaje. Estas intervenciones presentaron resultados favorables y efectos positivos en el desarrollo infantil.

Sin embargo, se observó una gran heterogeneidad entre las intervenciones analizadas, con variaciones en el contenido, el formato y la duración, además de la escasez de información sobre los materiales y estrategias utilizados. Esta falta de estandarización dificulta la identificación de lo que es más eficaz, lo que limita la replicación y debilita la base empírica para las políticas públicas.

El mapeo de las intervenciones realizadas con cuidadores de niños institucionalizados responde parcialmente a la necesidad de identificar características relevantes para la elaboración de futuros programas de intervención para la promoción del desarrollo infantil. En este sentido, se sugiere la realización de más estudios que proporcionen evidencia sólida sobre las características y los resultados de las intervenciones realizadas con cuidadores de niños institucionalizados para la promoción del desarrollo infantil, con el fin de orientar la práctica. Cabe destacar, además, la necesidad de elaborar e implementar gradualmente intervenciones de alta calidad, sostenibles y replicables con cuidadores de niños institucionalizados, con el fin de fortalecer las estructuras institucionales y ayudar a que los niños que viven en estos espacios tengan mayores posibilidades de alcanzar su máximo potencial de desarrollo.

## Data Availability

Todo el conjunto de datos que respalda los resultados de este estudio se ha publicado en el propio artículo. Se han extraído de artículos y fuentes disponibles públicamente, tal y como se detalla en el método, y están disponibles en la tabla de estudios incluida en la revisión.
